# Analytic study and statistical enforcement of extended beta functions imposed by Mittag-Leffler and Hurwitz-Lerch Zeta functions

**DOI:** 10.1016/j.mex.2025.103206

**Published:** 2025-02-13

**Authors:** Faten F. Abdulnabi, Hiba F. Al-Janaby, Firas Ghanim

**Affiliations:** a Department of Mathematics, College of Science, University of Baghdad, Iraq; b Ministry of Education, Al-Rusafa 2, Baghdad 10082, Iraq; c Department of Mathematics, College of Sciences, University of Sharjah, United Arab Emirates

**Keywords:** Special functions, Gamma function, Beta function, Hurwitz-Lerch zeta type function, Mellin transforms, Beta distribution, fractional derivative operator, This paper presents notable progress in Special Function Theory and its applications, broadening the use of Beta functions in both pure and practical mathematics, including fractional calculus and statistical distribution modeling

## Abstract

Special Function Theory is used in many mathematical fields to model scientific progress, from theoretical to practical. This helps efficiently analyze the newly expanded Beta class of functions on a complicated domain. We use Mittag-Leffler and Hurwitz Lerch zeta (HLZ) kernels to produce the Beta function using the convolution tool. This special function advances a statistical implementation research approach. This unique function also discusses and gives analytical benefits, including functional and summation relations, Mellin transformations, and integral representations. Additionally, many derivative formulae are obtained. The statistical implementation of expanded Beta distribution using the suggested beta function was also conducted. We use the extended Beta function to create the new extended ordinary hypergeometric function and Kummer function. Derivative formulae, integral representations, generating functions, and fractional derivatives are also investigated.

• Developed utilizing Mittag-Leffler and Hurwitz Lerch Zeta functions as kernels, delivering increased analytical and computational capabilities.
• Comprises derivative formulae, integral representations, Mellin transformations, and generating functions, offering a comprehensive mathematical foundation.
• Illustrates the use of the extended Beta function inside the Beta distribution, highlighting its statistical importance.

## Graphical abstract

**Figure:**
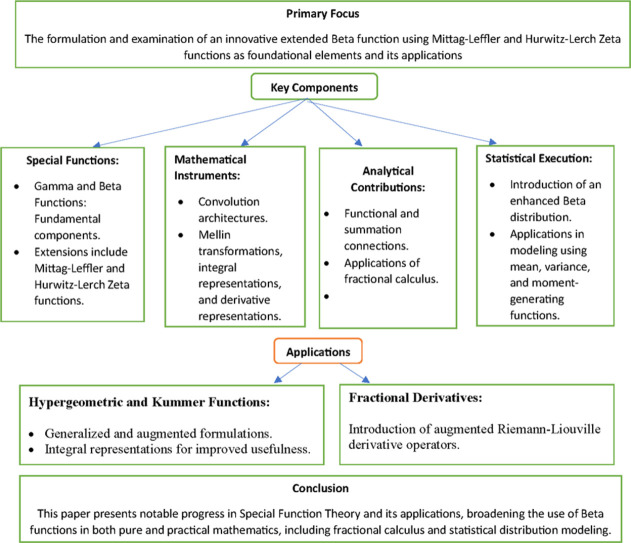

Specifications table

**Table:** 

Subject area:	Mathematics and Statistics
More specific subject area:	*Special functions; Gamma function; Beta function; Hurwitz-Lerch zeta type function; Mellin transforms; Beta distribution; fractional derivative operator.*
Name of your method:	*This paper presents notable progress in Special Function Theory and its applications, broadening the use of Beta functions in both pure and practical mathematics, including fractional calculus and statistical distribution modeling.*
Name and reference of original method:	*fractional calculus and statistical distribution modeling.*

## Method details

In mathematics, complex analysis discusses the analytic functions of complex numbers. The term complex numbers acquire extensive utilization in various realms of mathematics, especially in functional analysis, see [[1], [2], [3], [4]]. The theory of special function (SFT) is a prominent and captivating theme in complex analysis. Special Functions (SFs) are created by solving numerous partial differential equations and physical problems, which are usually generalized formulas of elementary functions. At that point, SFs are mathematical functions that act in complex domains, have private merits, and are often named after the initials of scientists who innovated them. Notable examples include the hypergeometric function, Wright function, Mittag-Leffler function, Meijer G-function, Hurwitz zeta function, and others. Remarkably, diverse representations of SFs, such as power series (special series) or integral representations, are utilized in mathematical evaluation. This appeals in mathematical processes to create SFs, namely convolution (Hadamard) product and symbolized by *. It was first posed by Hadamard as [5]: for analytical power series $\omega_{1}\left(z\right)=\sum_{\kappa =0}^{\infty }\rho_{\kappa ,1}\;z^{\kappa }$ and $\omega_{2}\left(z\right)=\sum_{\kappa =0}^{\infty }\rho_{\kappa ,2}\;z^{\kappa }\;$ in consecutive disks $\Delta_{1}$ and $\Delta_{2}$ centered at the origin, the convolution product of $\omega_{1}$ and $\omega_{2}$, represented by $\theta_{1}*\theta_{2}$, produces a third power series expressed as:

(1) $$\left(\omega_{1}*\omega_{2}\right)\left(z\right)=\sum_{\kappa =0}^{\infty }\rho_{\kappa ,1}\;\rho_{\kappa ,2}\;z^{\kappa }\;.$$

The vital idea of SFs lies in the effect of their complex parameters and variables in resolving and interpreting numerous scientific problems. Therefore, SFs are essential in developing ongoing investigations. Interestingly, Special Function Theory (SFT) is closely connected to Geometric Function Theory (GFT), which explores the geometric merits of analytical functions acting in a complex open unit disk. In this context, several investigators contributed greatly to its development, see ([6,7]). Indeed, the intensive study of SFs has occupied a major position in the scientific disciplines of applied mathematics; one can be referred to ([8,9]). The cornerstone of SFT is the Gamma function (Euler Integral of the second kind). Historically, the study of the Gamma function originally stemmed from a letter written by Euler to Goldbach in 1729, which is a more extended and generalized factorial function. It was rendered as [10]:

(2) $$\Gamma \left(\xi \right)=\int_{0}^{\infty }{℘}^{\xi -1}\;e^{-℘}\;d℘,\;\left(R\left(℘\right)>0\right).$$

where is the analytic function in R(℘)>0. This function, as stated in (2), has attracted the interest of some researchers, such as Legendre and Gauss. In terms of (2), Pochhammer constructed a rising function, indicated by Pochhammer's symbol, as [10]:

(3) $${\left(\xi \right)}_{\kappa }=\frac{\Gamma \left(\xi +\kappa \right)}{\Gamma \left(\xi \right)}=\left\{ \begin{array}{c} \xi \left(\xi +1\right)\ldots \left(\xi +\kappa -1\right),\;\left(\kappa \in N;\xi \in C\setminus \;\left\{0\right\}\right)\\ 1,\;\left(\kappa =0\right). \end{array}\right.$$

It plays a leading role in exploring many SFs, one of which is called Kummer functions. The class of Kummer function (confluent hypergeometric functions of the first kind) L(τ;ϱ;z) was first rendered by Kummer [11] in 1837 as:

(4) $${}_{1}\;F_{1}\left(\tau ;\varrho ;z\right)=L\left(\tau ;\varrho ;z\right)=\sum_{\kappa =0}^{\infty }\frac{{\left(\tau \right)}_{\kappa }}{{\left(\varrho \right)}_{\kappa }}\;\frac{\;z^{\kappa }}{\kappa !}\;=\frac{\Gamma \left(\varrho \right)}{\Gamma \left(\tau \right)}\sum_{\kappa =0}^{\infty }\;\frac{\Gamma \left(\tau +\kappa \right)}{\;\Gamma \left(\varrho +\kappa \right)}\;\frac{\;z^{\kappa }}{\kappa !}.$$

where τ,ϱ∈C,ϱ≠0,−1,−2,… and z∈C. This function is a resolution to the following Kummer's differential equation

(5) $$z\;\frac{d^{2}℘}{dz^{2}}+\left(\tau -z\right)\frac{d℘}{dz}-\tau \;d℘=0,$$

The Kummer equation is a significant differential equation employed in various disciplines of engineering, chemistry, and physics [11]. The underlying cause for this significance is that many of the SFs of mathematical physics are expressed based on Kummer functions. The authors have continued the study of new classes of Kummer functions, along with their modifications, generalizations, and extensions, see [[12], [13], [14], [15]].

More generally, the following Gaussian (ordinary) hypergeometric function is posed as [16]:

(6) $${}_{2}\;F_{1}\left(\tau ,\varsigma ;\varrho ;z\right)=\sum_{\kappa =0}^{\infty }\frac{{\left(\tau \right)}_{\kappa }\;{\left(\varsigma \right)}_{\kappa }}{{\left(\varrho \right)}_{\kappa }}\;\frac{\;z^{\kappa }}{\kappa !}\;.$$

where τ,ς,ϱ∈C,ϱ≠0,−1,−2,…, and |z|<1, This function achieves the following second-order hypergeometric differential equation:

(7) $$z\left(1-z\right)\;\frac{d^{2}℘}{dz^{2}}+\left[\varrho -\left(\tau +\varsigma +1\right)\right]\frac{d℘}{dz}-\tau \varsigma \;d℘=0.$$

The hypergeometric function is a generalized formula of the geometric series ${}_{2}^{\;}F_{1}\left(1,\varsigma ;\varsigma ;z\right)=\sum_{\kappa =0}^{\infty }\;z^{\kappa }\;$. Further, the Kummer function given in (4) can be designated as a limit of the hypergeometric function: $L\left(\tau ;\varrho ;z\right)=\lim_{z\to \infty }{}_{2}^{\;}F_{1}\left(\tau ,\varsigma ;\varrho ;\varsigma^{-1}z\right)$. Pursuing this thread of study, numerous researchers attempted to discover new classes of hypergeometric functions and have highlighted many new aspects of this area. In other words, the theory of hypergeometric functions is considered the most paramount and effective theme in several domains of mathematics. Specifically, the relevant role played by hypergeometric functions in GFT by resolving a major problem called "Bieberbach conjecture", is well known [17]. This theory was first studied by the infamous scholar Euler. The methodical study of hypergeometric functions began by Gauss in 1813, followed by the renowned scholars Kummer in 1836 and Riemann in 1857 (for details, see [16]). Since then, the hypergeometric functions have come under various formulas and have had several fruitful implementations. Works that have addressed this topic include those in ([18,19]).

In this regard, corresponding to Gamma function (2), Mittag Leffler [20] in 1903 presented the function $\;E_{\gamma }\left(z\right)$ in the following formula:

(8) $$\;E_{\gamma }\left(z\right)=\sum_{\kappa =0}^{\infty }\frac{z^{\kappa }}{\Gamma \left(\gamma \kappa +1\right)},\;\left(R\left(\gamma \right)>0,\;z\in C\right).$$

This one-parameter function is called a Mittag-Leffler function, which is a naturally generalized formula of exponential function $e^{z}$. Then, Wiman ([21,22]) in 1905 proposed a more general $\;E_{\gamma ,\varrho }\left(z\right)$ for (8) as:

(9) $$\;E_{\gamma ,\varrho }\left(z\right)=\sum_{\kappa =0}^{\infty }\frac{z^{\kappa }}{\Gamma \left(\gamma \kappa +\varrho \right)},\;\left(R\left(\gamma \right),\;R\left(\varrho \right)>0\right),$$

which is named the two-parameter Mittag-Leffler function (Wiman function). Since then, the attention to Mittag-Leffler type functions has increased greatly, primarily due to their effort for applicability in diverse reaction-diffusion problems and the emergence of a variety of generalized formulas in resolutions of fractional (differential and integral) equations ([23,24]).

Analogous to Pochhammer's symbol (3), a novel interesting class of Mittag-Leffler-Kummer functions was considered by Ghanim et al. [12] in 2024 as:

(10) $$EL_{\upsilon ,\varrho }^{\sigma ,\gamma }\left(z\right)=\sum_{\kappa =0}^{\infty }\frac{{\left(\gamma \right)}_{\sigma \kappa }}{{\left(\varrho \right)}_{\upsilon \kappa }}\;\frac{\;z^{\kappa }}{\kappa !}\;=\frac{\Gamma \left(\varrho \right)}{\Gamma \left(\gamma \right)}\sum_{\kappa =0}^{\infty }\;\frac{\Gamma \left(\sigma \kappa +\gamma \right)}{\;\Gamma \left(\upsilon \kappa +\varrho \right)}\;\frac{\;z^{\kappa }}{\kappa !},$$

(γ,ϱ,σ,υ∈C,ϱ≠0,−1,−2,…,z∈C),R(γ),R(ϱ),R(σ),R(υ)>0.

Obviously, the Mittag-Leffler function and Kummer function are connected by their identities: $EL_{\gamma ,\varrho }^{1,1}\left(z\right)=\;E_{\gamma ,\varrho }\left(z\right)$; $EL_{\gamma ,1}^{1,1}\left(z\right)=\;E_{\gamma }\left(z\right)$; $EL_{1,\varrho }^{1,\tau }\left(z\right)=L\left(\tau ;\varrho ;z\right)$.

Additionally, the effective aspect of SFs in the study of Analytic Number Theory (ANT) has given rise to the Hurwitz Lerch zeta (HL-Z) functions, indicated by HL(z,μ,η). This function is stated in [16,25] as:

(11) $$HL\left(\mu ,\eta ,z\right)=\sum_{\kappa =0}^{\infty }\frac{z^{\kappa }}{{\left(\kappa +\eta \right)}^{\mu }},$$

where, μ∈C, $\eta \in C\setminus Z_{0}^{-}$, when |z|<1 and R(μ)>1, when |z|=1. This function continues meromorphically to C, (exclude for a simple pole at t=1 along with residue 1). Following that, Goyal and Laddha [26] in 1997 provided a more general HL-Z function:

(12) $$HL^{*}\left(\tau ,\mu ,\eta ,z\right)=\sum_{\kappa =0}^{\infty }\frac{{\left(\tau \right)}_{\kappa }}{\kappa !}\;\frac{z^{\kappa }}{{\left(\kappa +\eta \right)}^{\mu }},$$

where, μ,τ∈C, $\eta \in C\setminus Z_{0}^{-}$, when |z|<1, and R(μ−τ)>1, when |z|=1. Afterward, prominent authors offered a variety of appealing studies that involved HL-Z functions ([27,28]).

In this regard, one of the great functions in SFT is the Beta function, also termed Euler's Integral of the first kind, which has major roles in developing scientific research, such as engineering and mathematics. It is formulated as [10]:

(13) $$B\left(\xi_{1},\xi_{2}\right)=\;\int_{0}^{1}{℘}^{\xi_{1}-1}\;{\left(1-℘\right)}^{\xi_{2}-1}\;d℘,\;\left(\;R\left(\xi_{1}\right),\;R\left(\xi_{2}\right)>0\right).$$

Besides, closely connected to the Beta function is the Gamma function given by (2) as [10]:

(14) $$B\left(\xi_{1},\xi_{2}\right)=\frac{\Gamma \left(\xi_{1}+\xi_{2}\right)}{\Gamma \left(\xi_{1}\right)\;\Gamma \left(\xi_{2}\right)}\;.$$

In the 1990s, the study of extended Gamma and Beta functions became a catalyst theme of scientific research that attracted investigators. The extended Gamma function (2) was first presented by Chaudhry and Zubair [29] in 1994 as follows:

(15) $$\Gamma_{\delta }\left(\xi \right)=\int_{0}^{\infty }{℘}^{\xi -1}\;e^{-℘-\delta ℘}\;d℘,\;\left(R\left(℘\right),\;\delta >0\right).\;$$

Importantly, the role played by special classes of analytic functions, especially exponential, Kummer, and Mittag-Leffler functions, as kernels is of great significance for rendering new versions of extended Beta functions. In 1997, Chaudhry et al. [30] postulated the first extended Beta function that includes the exponential function as kernel, as follows:

(16) $$B_{\delta }\left(\xi_{1},\xi_{2}\right)=\int_{0}^{1}{℘}^{\xi_{1}-1}\;{\left(1-℘\right)}^{\xi_{2}-1}\;e^{\frac{-\delta }{℘\left(1-℘\right)}}\;d℘,\;\left(R\left(\xi_{1}\right),\;R\left(\xi_{2}\right),\;R\left(\delta \right)>0\right).$$

For δ=0 in (16), it turns to $B(\xi_{1},\xi_{2})$ given in (13). Later, in 2011, Özergin et al. [31] suggested and analyzed the more general extended Beta function based on (4) as a kernel. It is instituted as:

(17) $$B_{\delta ,\varrho }^{\tau }\left(\xi_{1},\xi_{2}\right)=\int_{0}^{1}{℘}^{\xi_{1}-1}{\left(1-℘\right)}^{\xi_{2}-1}{\;}_{1}F_{1}\left(\tau ;\varrho ;\frac{-\delta }{℘\left(1-℘\right)}\right)d℘,$$

$$\left(\min\left\{R\left(\xi_{1}\right),\;R\left(\xi_{2}\right),\;R\left(\gamma \right),\;R\left(\varrho \right),\;R\left(\delta \right)\right\}>0\;\right).$$

Obviously, for τ=ϱ in (17), it yields to $B_{\delta }\left(\xi_{1},\xi_{2}\right)$ given by (16). If δ=0 and τ=ϱ in (17), it reduces to $B(\xi_{1},\xi_{2})$ written by (13).

In 2017, Pucheta [32] discussed the different extended Beta functions in terms of (8) as a kernel given as:

(18) $$B_{\delta }^{\gamma }\left(\xi_{1},\;\xi_{2}\right)=\int_{0}^{\infty }{℘}^{\xi_{1}-1}{\left(1-℘\right)}^{\xi_{2}-1}\;E_{\gamma }\left(-\delta ℘\left(1-℘\right)\right)d℘,$$

$$\left(\min\left\{R\left(\xi_{1}\right),\;R\left(\xi_{1}\right),\;R\left(\gamma \right),\;R\left(\delta \right)\right\}>0\;\right).$$

Notice that for δ=0 and γ=1 in (18), it gains to $B({℘}_{1},\;{℘}_{2})$ defined by (13). Analogous to Pucheta, Shadab et al. [33] in 2018 presented another extended Beta function by employing (8) as a kernel. It is stated as follows:

(19) $$B_{\delta }^{\gamma }\left(\xi_{1},\;\xi_{2}\right)=\int_{0}^{1}{℘}^{\xi_{1}-1}{\left(1-℘\right)}^{\xi_{2}-1}\;\;E_{\gamma }\left(-\delta {℘}^{-1}{\left(1-℘\right)}^{-1}\right)d℘,$$

$$\left(\min\left\{R\left(\xi_{1}\right),\;R\left(\xi_{1}\right),\;R\left(\gamma \right),\;R\left(\delta \right)\right\}>0\;\right).$$

For γ=1 in (19), it achieves to $B_{\delta }\left(\xi_{1},\xi_{2}\right)$ given by (16). If δ=0 and γ=1 in (19), it acquires to $B(\xi_{1},\xi_{2})$ expressed by (13).

In 2021, Goyal et al. [34] investigated the extended Beta function by utilizing (9) as a kernel. It is coined as:

(20) $$B_{\delta }^{\left(\gamma ;\varrho \right)}\left(\xi_{1},\;\xi_{2}\right)=\int_{0}^{1}{℘}^{\xi_{1}-1}{\left(1-℘\right)}^{\xi_{2}-1}\;E_{\gamma ,\varrho }\left(-\delta {\left(℘\left(1-℘\right)\right)}^{-1}\right)d℘,$$

$$\left(\min\left\{R\left(\xi_{1}\right),\;R\left(\xi_{2}\right),\;R\left(\gamma \right),\;R\left(\varrho \right),\;R\left(\delta \right)\right\}>0\;\right).$$

Evidently, for ϱ=1 in (20), it turns to $B_{\delta }^{\gamma }\left(\xi_{1},\;\xi_{2}\right)$ written by (19). If γ=ϱ=1 in (20), it reduces to $B_{\delta }\left(\xi_{1},\xi_{2}\right)$ given by (16). Also, if δ=0 and γ=ϱ=1 in (20), it arrives to $B(\xi_{1},\;\xi_{2})$ posed in (13).

In 2024, Ghanim et al. [35] presented a new extended Beta function called Euler's Beta Mittag-Leffler-Kummer function by employing (10) as a kernel. It is formulated as:

(21) $$B_{\delta ,\upsilon ,\varrho }^{\left(\sigma ,\gamma \right)}\left(\xi_{1},\;\xi_{2}\right)=\int_{0}^{1}{℘}^{\xi_{1}-1}{\left(1-℘\right)}^{\xi_{2}-1}\;EL_{\upsilon ,\varrho }^{\sigma ,\gamma }\left(-\delta {\left(℘\left(1-℘\right)\right)}^{-1}\right)d℘,$$

$$\left(\min\left\{R\left(\xi_{1}\right),\;R\left(\xi_{2}\right),\;R\left(\gamma \right),\;R\left(\varrho \right),\;R\left(\delta \right)\right\}>0\;\right).$$

For σ=γ=1 in (21), it turns to $\Gamma \left(\varrho \right)B_{\delta }^{\left(\gamma ;\varrho \right)}\left(\xi_{1},\;\xi_{2}\right)$ given in (15). If σ=γ=ϱ=1 in (21), it reduces to $B_{\delta }^{\gamma }\left(\xi_{1},\;\xi_{2}\right)$ posed in (19). If σ=γ=ϱ=υ=1 in (21), it yields to $B_{\delta }\left(\xi_{1},\xi_{2}\right)$ written in (16). Further, if σ=γ=ϱ=υ=1 and δ=0 in (21), it reduces to $B(\xi_{1},\xi_{2})$ given in (13).

In mathematics, the realm of Fractional Calculus (FC) has played a remarkable role and is considered a dynamic tool for modeling and solving manifold problems in engineering, combined with pure and applied areas of sciences, see [[36], [37], [38]]. It was systematically developed in the 19th century. This realm deals with arbitrary (real or complex) order of integration and differentiation operations and is the generalized formula of classical calculus. The idea of FC was first put forward by legendary scientists in a letter from L'Hopital addressed to Leibniz in 1695, in which the question of the sense of the semi-derivative was raised. Subsequently, several great mathematicians, such as Euler, Riemann, Liouville, Fourier, Laplace, and others, contributed significantly to the evolution of FC. In this line, SFT is considered the cornerstone of the theoretical formulation of FC, which led to its emergence and evolution. In other words, the theoretical construction of fractional (integral and derivative) operators is often characterized in terms of the Gamma function written in (2). Prominent examples of this include the traditional Riemann-Liouville (RL) fractional derivative of ω(z) of order q formulated as [39]:

$$D_{z}^{q}\omega \left(z\right)=\frac{1}{\Gamma \left(-q\right)}\int_{0}^{z}{\left(z-℘\right)}^{-q-1}\;\omega \left(℘\right)\;d℘,$$

where R(q)<0. For R(q)≥0, consider l∈N such that l−1≤R(q)<l. Then, RL fractional derivative of ω(z) of order q is expressed as:

$$D_{z}^{q}\omega \left(z\right)=\frac{d^{l}}{dz^{l}}D_{z}^{q-l}\omega \left(z\right)=\frac{d^{l}}{dz^{l}}\left\{\frac{1}{\Gamma \left(l-q\right)}\int_{0}^{z}{\left(z-℘\right)}^{l-q-1}\;\omega \left(℘\right)\;d℘\right\}.$$

Then, obtaining formulas of RL-type fractional derivatives correlated with a variety of SFs of interest is currently investigated; see ([40,41]). By pursuing the interesting previous studies in developing the theme of extended Beta functions, this sequel continues to present an analysis by using the eminent SFs called generalization of Mittag-Leffler and Hurwitz Lerch zeta functions as kernel to render new extended Beta functions. This study introduces a new generalized Beta function in terms of the Mittag-Leffler function (9) and HL-Z function (12) utilizing a convolution structure. Moreover, diverse analytical merits of this posed Beta function are provided, including functional and summation relations, Mellin transforms, integral representations, and assorted derivative formulas. Furthermore, the statistical implementation of the extended Beta distribution is also achieved.

In this paper, our approach for constructing extended Beta functions explicitly integrates Mittag-Leffler and Hurwitz-Lerch Zeta (HLZ) functions as kernels, using the convolution technique to get an extended Beta function. This dual-function structure improves analytical depth and flexibility, enabling multi-parameter generalizations and broader applicability. This research advances the statistical implementation by applying the suggested Beta function to the enlarged Beta distribution. Accordingly, it gives a comprehensive framework for statistical modeling, improving applications in probability theory and statistical science. Our methodology presents novel extended ordinary hypergeometric functions and extended Kummer functions, which are derived from the suggested Beta function. These contributions address a deficiency in the current literature by extending these functions within a cohesive and generalized approach. This effort improves upon previous endeavors by obtaining fractional derivatives of the expanded Beta function. Integral representations and generation functions are introduced to improve the theoretical context of these functions. The resources that these improvements provide are substantial and can be used to address higher-order differential equations and series expansions. For more details see [39,42] and [43].

## Method validation

Proposed Beta- Mittag-Leffler-Hurwitz-Lerch Zeta Function $B_{\delta ,\varrho ,\eta }^{\tau ,\mu }\left(\xi_{1},\;\xi_{2}\right)$

This section presents a new extended Beta function, indicated by $B_{\delta ,\varrho ,\eta }^{\tau ,\mu }\left(\xi_{1},\;\xi_{2}\right)$, related to the Mittag-Leffler (9) and the HL-Z (12) functions using the convolution principle (1).

The Mittag-Leffler function (Wiman's function) is expressed in (9) as:

$$\;E_{\gamma ,\varrho }\left(z\right)=\sum_{\kappa =0}^{\infty }\frac{z^{\kappa }}{\Gamma \left(\kappa \gamma +\varrho \right)},\;\left(R\left(\gamma \right),\;R\left(\varrho \right)>0\right).$$

For γ=1, the function $\;E_{\gamma ,\varrho }\left(z\right)$ turns to

(22) $$\;E_{1,\varrho }\left(z\right)=\sum_{\kappa =0}^{\infty }\frac{z^{\kappa }}{\Gamma \left(\kappa +\varrho \right)},\;\left(R\left(\varrho \right)>0\right).$$

Furthermore, the HL-Z function $HL^{*}\left(z,\tau ,\mu ,\eta \right)$ is stated in (12) as:

$$HL^{*}\left(,\tau ,\mu ,\eta ,z\right)=\sum_{\kappa =0}^{\infty }\frac{{\left(\tau \right)}_{\kappa }}{\kappa !}\;\frac{z^{\kappa }}{{\left(\kappa +\eta \right)}^{\mu }},$$

(μ,τ∈C, $\eta \in C\setminus Z_{0}^{-}$, when |z|<1, R(μ)>1, when |z|=1).

Thus, from (1), (22), and (12), the following new function is acquired:

(23) $$\;\psi_{\varrho ,\eta }^{\tau ,\mu }\left(z\right)=HL^{*}\left(\tau ,\mu ,\eta ,z\right)*\Gamma \left(\varrho \right)\;E_{1,\varrho }\left(z\right)=\sum_{\kappa =0}^{\infty }\;\frac{{\left(\tau \right)}_{\kappa }}{\;{\left(\varrho \right)}_{\kappa }{\left(\kappa +\eta \right)}^{\mu }}\;\frac{\;z^{\kappa }\;}{\;\kappa !\;}\;.$$

**Remark 1.** For the applicable parameters, τ,μ and ϱ, the function $\psi_{\varrho ,\eta }^{\tau ,\mu }\left(z\right)$ written in (23) reduces to specified functions. Hence, the following special cases are yielded:

1. $\psi_{1,\eta }{}^{1,0}\left(z\right)=e^{z},$
2. $\psi_{\varrho ,\eta }{}^{\tau ,0}\left(z\right)={}_{1}^{F_{1}}\left(\tau ;\varrho ;z\right)$ given in (4).

Based on Eq. (23), the new extended Beta function $B_{\delta ,\varrho ,\eta }^{\tau ,\mu }\left(\xi_{1},\;\xi_{2}\right)$ is expressed by the following definition.

**Definition 1.** For δ≥0, the extended Beta function $B_{\delta ,\varrho ,\eta }^{\tau ,\mu }\left(\xi_{1},\;\xi_{2}\right)$ is formulated as:

(24) $$B_{\delta ,\varrho ,\eta }^{\tau ,\mu }\left(\xi_{1},\;\xi_{2}\right)=\int_{0}^{1}{℘}^{\xi_{1}-1}{\left(1-℘\right)}^{\xi_{2}-1}\;\psi_{\varrho ,\eta }^{\tau ,\mu }\left(-\delta {\left(℘\left(1-℘\right)\right)}^{-1}\right)d℘,$$

where $\min\{R\left(\xi_{1}\right),\;R\left(\xi_{2}\right),\;R\left(\tau \right),\;R\left(\mu \right),\;R\left(\varrho \right),\;R\left(\eta \right)\}>0\;$.

**Remark 2.** The noteworthy special cases of extended Beta function $B_{\delta ,\varrho ,\eta }^{\tau ,\mu }\left(\xi_{1},\;\xi_{2}\right)$ are:

1. $B_{\delta ,\varrho ,\eta }^{\tau ,0}\left(\xi_{1},\;\xi_{2}\right)=B_{\delta ,\varrho }^{\tau }\left(\xi_{1},\xi_{2}\right)$ stated in (17),
2. $B_{\delta ,1,\eta }^{1,0}\left(\xi_{1},\;\xi_{2}\right)=\;B_{\delta }\left(\xi_{1},\xi_{2}\right)\;$given by (16),
3. $B_{0,1,\eta }^{1,0}\left(\xi_{1},\;\xi_{2}\right)=\;B\left(\xi_{1},\xi_{2}\right)\;$written by (13).

### Analytical Merits of $B_{\delta ,\varrho ,\eta }^{\tau ,\mu }\left(\xi_{1},\;\xi_{2}\right)$

In this section, several analytical merits of the new extended Beta function $B_{\delta ,\varrho ,\eta }^{\tau ,\mu }\left(\xi_{1},\;\xi_{2}\right)$ in (24) are investigated, which involve functional and summation relations, Mellin transforms, and integral representations. Furthermore, assorted derivative formulas and the statistical implementation of the extended Beta distribution are also attained.

Theorem 1 *For*δ≥0*, and*$\min\{R\left(\xi_{1}\right),\;R\left(\xi_{2}\right),\;R\left(\tau \right),\;R\left(\mu \right),\;R\left(\varrho \right),\;R\left(\eta \right)\}>0\;$*, then the extended Beta function*$B_{\delta ,\varrho ,\eta }^{\tau ,\mu }\left(\xi_{1},\;\xi_{2}\right)$*in (23) attains the symmetric relation*.

(25) $$B_{\delta ,\varrho ,\eta }^{\tau ,\mu }\left(\xi_{1},\;\xi_{2}\right)=B_{\delta ,\varrho ,\eta }^{\tau ,\mu }\left(\;\xi_{2},\xi_{1}\right).$$

Proof. From Eq. (24), we acquire

$$B_{\delta ,\varrho ,\eta }^{\tau ,\mu }\left(\xi_{1},\;\xi_{2}\right)=\int_{0}^{1}{℘}^{\xi_{1}-1}{\left(1-℘\right)}^{\xi_{2}-1}\;\psi_{\varrho ,\eta }^{\tau ,\mu }\left(-\delta {\left(℘\left(1-℘\right)\right)}^{-1}\right)d℘.$$

*By setting* ℘=(1−ϰ) *in the above equation, it leads to*

$$B_{\delta ,\varrho ,\eta }^{\tau ,\mu }\left(\xi_{1},\;\xi_{2}\right)=\int_{0}^{1}{\left(1-\varkappa \right)}^{\xi_{1}-1}\;\varkappa^{\xi_{2}-1}\;\psi_{\varrho ,\eta }^{\tau ,\mu }\left(-\delta {\left(\varkappa \left(1-\varkappa \right)\right)}^{-1}\right)d\varkappa \;.$$

*Therefore, by* Eq. (24) *, we gain*

$$B_{\delta ,\varrho ,\eta }^{\tau ,\mu }\left(\xi_{1},\;\xi_{2}\right)=B_{\delta ,\varrho ,\eta }^{\tau ,\mu }\left(\xi_{2},\xi_{1}\right).$$

Theorem 2 *For*δ≥0*, and*$\min\{R\left(\xi_{1}\right),\;R\left(\xi_{2}\right),\;R\left(\tau \right),\;R\left(\mu \right),\;R\left(\varrho \right),\;R\left(\eta \right)\}>0$*, then the extended Beta function*$B_{\delta ,\varrho ,\eta }^{\tau ,\mu }\left(\xi_{1},\;\xi_{2}\right)$*in (24) achieves the functional relation*.

(26) $$B_{\delta ,\varrho ,\eta }^{\tau ,\mu }\left(\xi_{1},\;\xi_{2}\right)=B_{\delta ,\varrho ,\eta }^{\tau ,\mu }\left(\xi_{1},\;\xi_{2}+1\right)+B_{\delta ,\varrho ,\eta }^{\tau ,\mu }\left(\xi_{1}+1,\;\xi_{2}\right).$$

*Proof. By utilizing* Eq. (24) *and the right of* Eq. (26) *, we yield*

$$B_{\delta ,\varrho ,\eta }^{\tau ,\mu }\left(\xi_{1},\;\xi_{2}+1\right)+B_{\delta ,\varrho ,\eta }^{\tau ,\mu }\left(\xi_{1}+1,\;\xi_{2}\right)=$$

$$=\int_{0}^{1}{℘}^{\xi_{1}-1}{\left(1-℘\right)}^{\xi_{2}}\;\psi_{\varrho ,\eta }^{\tau ,\mu }\left(-\delta {\left(℘\left(1-℘\right)\right)}^{-1}\right)d℘+\int_{0}^{1}{℘}^{\xi_{1}}{\left(1-℘\right)}^{\xi_{2}-1}\;\psi_{\varrho ,\eta }^{\tau ,\mu }\left(-\delta {\left(℘\left(1-℘\right)\right)}^{-1}\right)d℘$$

$$=\int_{0}^{1}\left[{℘}^{-1}+{\left(1-℘\right)}^{-1}\right]\;{℘}^{\xi_{1}}{\left(1-℘\right)}^{\xi_{2}}\;\psi_{\varrho ,\eta }^{\tau ,\mu }\left(-\delta {\left(℘\left(1-℘\right)\right)}^{-1}\right)d℘$$

$$=\int_{0}^{1}{℘}^{\xi_{1}-1}{\left(1-℘\right)}^{\xi_{2}-1}\;\psi_{\varrho ,\eta }^{\tau ,\mu }\left(-\delta {\left(℘\left(1-℘\right)\right)}^{-1}\right)d℘=B_{\delta ,\varrho ,\eta }^{\tau ,\mu }\left(\xi_{1},\;\xi_{2}\right).$$

Theorem 3 *For*δ≥0*, and*$\min\{R\left(\xi_{1}\right),\;R\left(\xi_{2}\right),\;R\left(\tau \right),\;R\left(\mu \right),\;R\left(\varrho \right),\;R\left(\eta \right)\}>0$*, then the extended Beta function*$B_{\delta ,\varrho ,\eta }^{\tau ,\mu }\left(\xi_{1},\;\xi_{2}\right)$*in (24) attains the summation relation*.

(27) $$B_{\delta ,\varrho ,\eta }^{\tau ,\mu }\left(\xi_{1},\;\xi_{2}\right)=\sum_{\kappa =0}^{\infty }B_{\delta ,\varrho ,\eta }^{\tau ,\mu }\left(\xi_{1}+\kappa ,\;\xi_{2}+1\right).$$

*Proof. From* Eq. (24) *and* ${\left(1-℘\right)}^{-1}=\sum_{\kappa =0}^{\infty }{℘}^{\kappa }$ *,* |℘|<1 *, we yield*

$$B_{\delta ,\varrho ,\eta }^{\tau ,\mu }\left(\xi_{1},\;\xi_{2}\right)=\int_{0}^{1}{℘}^{\xi_{1}-1}{\left(1-℘\right)}^{\xi_{2}-1}\;\psi_{\varrho ,\eta }^{\tau ,\mu }\left(-\delta {\left(℘\left(1-℘\right)\right)}^{-1}\right)d℘$$

$$=\int_{0}^{1}{℘}^{\xi_{1}-1}{\left(1-℘\right)}^{\xi_{2}}\;\sum_{\kappa =0}^{\infty }{℘}^{\kappa }\;\psi_{\varrho ,\eta }^{\tau ,\mu }\left(-\delta {\left(℘\left(1-℘\right)\right)}^{-1}\right)d℘$$

$$=\sum_{\kappa =0}^{\infty }\int_{0}^{1}{℘}^{\xi_{1}+\kappa -1}{\left(1-℘\right)}^{\xi_{2}}\;\psi_{\varrho ,\eta }^{\tau ,\mu }\left(-\delta {\left(℘\left(1-℘\right)\right)}^{-1}\right)d℘=\sum_{\kappa =0}^{\infty }B_{\delta ,\varrho ,\eta }^{\tau ,\mu }\left(\xi_{1}+\kappa ,\;\xi_{2}+1\right).$$

Theorem 4 *For*δ≥0*, and*$\min\{R\left(\xi_{1}\right),\;R\left(\xi_{2}\right),\;R\left(\tau \right),\;R\left(\mu \right),\;R\left(\varrho \right),\;R\left(\eta \right)\}>0$*, then the extended Beta function*$B_{\delta ,\varrho ,\eta }^{\tau ,\mu }\left(\xi_{1},\;\xi_{2}\right)$*in (24) obtains the summation relation*.

(28) $$B_{\delta ,\varrho ,\eta }^{\tau ,\mu }\left(\xi_{1},1-\;\xi_{2}\right)=\sum_{\kappa =0}^{\infty }\frac{\;{\left(\xi_{2}\right)}_{\kappa }\;}{\kappa !}\;B_{\delta ,\varrho ,\eta }^{\tau ,\mu }\left(\xi_{1}+\kappa ,\;1\right).$$

*Proof. From* Eq. (24) *and binomial expansion* ${\left(1-℘\right)}^{-\xi_{2}}=\sum_{\kappa =0}^{\infty }\frac{{\left(\xi_{2}\right)}_{\kappa }}{\;\kappa !\;}{℘}^{\kappa }$ *,* |℘|<1 *, we gain*

$$B_{\delta ,\varrho ,\eta }^{\tau ,\mu }\left(\xi_{1},1-\;\xi_{2}\right)=\int_{0}^{1}{℘}^{\xi_{1}-1}{\left(1-℘\right)}^{-\xi_{2}}\;\psi_{\varrho ,\eta }^{\tau ,\mu }\left(-\delta {\left(℘\left(1-℘\right)\right)}^{-1}\right)d℘$$

$$=\sum_{\kappa =0}^{\infty }\frac{{\left(\xi_{2}\right)}_{\kappa }}{\;\kappa !\;}\;\int_{0}^{1}{℘}^{\xi_{1}+\kappa -1}\;\psi_{\varrho ,\eta }^{\tau ,\mu }\left(-\delta {\left(℘\left(1-℘\right)\right)}^{-1}\right)\;d℘=\sum_{\kappa =0}^{\infty }\frac{\;{\left(\xi_{2}\right)}_{\kappa }\;}{\kappa !}\;B_{\delta ,\varrho ,\eta }^{\tau ,\mu }\left(\xi_{1}+\kappa ,\;1\right)=B_{\delta ,\varrho ,\eta }^{\tau ,\mu }\left(\xi_{1},1-\;\xi_{2}\right).$$

Theorem 5 *For*δδ≥0*, and*$\min\{R\left(\xi_{1}\right),\;R\left(\xi_{2}\right),\;R\left(\tau \right),\;R\left(\varrho \right),\;R\left(\mu \right)\}>0$*, then the extended Beta function*$B_{\delta ,\varrho ,\eta }^{\tau ,\mu }\left(\xi_{1},\;\xi_{2}\right)$*in (24) is related to the Beta function stated in (5)*.

(29) $$B_{\delta ,\varrho ,\eta }^{\tau ,\mu }\left(\xi_{1},\;\xi_{2}\right)=\sum_{\kappa =0}^{\infty }\;\frac{{\left(\tau \right)}_{\kappa }\;{\left(-\delta \right)}^{\kappa }}{\;\;{\left(\varrho \right)}_{\kappa }\;\kappa !\;{\left(\kappa +\eta \right)}^{\mu }\;}\;B\left(\xi_{1}-\kappa ,\xi_{2}-\kappa \right).$$

*Proof. From* Eq. (22) *, alternative* $\psi_{\varrho ,\eta }^{\tau ,\mu }\left(z\right)$ *to (24), we acquire*

$$B_{\delta ,\varrho ,\eta }^{\tau ,\mu }\left(\xi_{1},\;\xi_{2}\right)=\int_{0}^{1}{℘}^{\xi_{1}-1}{\left(1-℘\right)}^{\xi_{2}-1}\;\sum_{\kappa =0}^{\infty }\frac{\;{\left(\tau \right)}_{\kappa }}{\;{\left(\varrho \right)}_{\kappa }\;}\;\frac{{\left(-\delta \right)}^{\kappa }}{\;\kappa !\;{\left(\kappa +\eta \right)}^{\mu }\;{℘}^{\kappa }\;{\left(1-℘\right)}^{\kappa }}d℘$$

$$=\sum_{\kappa =0}^{\infty }\frac{{\left(\tau \right)}_{\kappa }\;{\left(-\delta \right)}^{\kappa }}{\;\;{\left(\varrho \right)}_{\kappa }\;\kappa !\;{\left(\kappa +\eta \right)}^{\mu }\;}\int_{0}^{1}{℘}^{\xi_{1}-\kappa -1}{\left(1-℘\right)}^{\xi_{2}-\kappa -1}\;d℘$$

$$=\sum_{\kappa =0}^{\infty }\frac{{\left(\tau \right)}_{\kappa }\;{\left(-\delta \right)}^{\kappa }}{\;\;{\left(\varrho \right)}_{\kappa }\;\kappa !\;{\left(\kappa +\eta \right)}^{\mu }\;}\;B\left(\xi_{1}-\kappa ,\xi_{2}-\kappa \right).$$

Theorem 6 *For*δ≥0*, and*$\min\{R\left(\xi_{1}\right),\;R\left(\xi_{2}\right),\;R\left(\tau \right),\;R\left(\mu \right),\;R\left(\varrho \right),\;R\left(\eta \right)\}>0$*, then the Mellin Transform of the extended Beta function*$B_{\delta ,\varrho ,\eta }^{\tau ,\mu }\left(\xi_{1},\;\xi_{2}\right)\;$*in (24) is provided by*

(30) $$\int_{0}^{\infty }\delta^{\alpha -1}B_{\delta ,\varrho ,\eta }^{\tau ,\mu }\left(\xi_{1},\;\xi_{2}\right)\;d\delta =B\left(\xi_{1}+\alpha ,\xi_{2}+\alpha \right)\;l_{\varrho ,\eta }^{\tau ,\mu }\left(\alpha \right),$$

where $l_{\varrho ,\eta }^{\tau ,\mu }\left(\alpha \right)=\int_{0}^{\infty }\delta^{\alpha -1}\;\psi_{\varrho ,\eta }^{\tau ,\mu }\left(-\delta \right)d\delta$.

*Proof. Multiplying* Eq. (24) *by* $\delta^{\alpha -1}$ *and the path of integration relates* δ *from the limit* δ=0 *to* δ=∞ *, we yield*

$$\int_{0}^{\infty }\delta^{\alpha -1}B_{\delta ,\varrho ,\eta }^{\tau ,\mu }\left(\xi_{1},\;\xi_{2}\right)\;d\delta =\int_{0}^{\infty }\delta^{\alpha -1}\left(\int_{0}^{1}{℘}^{\xi_{1}-1}{\left(1-℘\right)}^{\xi_{2}-1}\;\psi_{\varrho ,\eta }^{\tau ,\mu }\left(-\delta {\left(℘\left(1-℘\right)\right)}^{-1}\right)d℘\right)\;d\delta$$

$$=\int_{0}^{1}{℘}^{\xi_{1}-1}{\left(1-℘\right)}^{\xi_{2}-1}\;\left(\int_{0}^{\infty }\delta^{\alpha -1}\;\psi_{\varrho ,\eta }^{\tau ,\mu }\left(-\delta {\left(℘\left(1-℘\right)\right)}^{-1}\right)d\delta \right)\;d℘$$

*Considering* $\nu =\delta {\left(℘(1-℘)\right)}^{-1},\;$ *we acquire*

$$\int_{0}^{\infty }\delta^{\alpha -1}B_{\delta ,\varrho ,\eta }^{\tau ,\mu }\left(\xi_{1},\;\xi_{2}\right)\;d\delta =\int_{0}^{1}{℘}^{\xi_{1}+\alpha -1}\;{\left(1-℘\right)}^{\xi_{2}+\alpha -1}\;\left(\int_{0}^{\infty }\nu^{\alpha -1}\;\psi_{\varrho ,\eta }^{\tau ,\mu }\left(-\nu \right)d\nu \right)\;d℘$$

$$=B\left(\xi_{1}+\alpha ,\xi_{2}+\alpha \right)\;l_{\varrho ,\eta }^{\tau ,\mu }\left(\alpha \right).$$

Theorem 7 *For*δ≥0*, and*$\min\{R\left(\xi_{1}\right),\;R\left(\xi_{2}\right),\;R\left(\tau \right),\;R\left(\mu \right),\;R\left(\varrho \right),\;R\left(\eta \right)\}>0$*, then the extended Beta function*$B_{\delta ,\varrho ,\eta }^{\tau ,\mu }\left(\xi_{1},\;\xi_{2}\right)$*in (24) has the integral representation*.

(31) $$B_{\delta ,\varrho ,\eta }^{\tau ,\mu }\left(\xi_{1},\;\xi_{2}\right)=2\int_{0}^{\frac{\pi }{2}}{\left(\cos\left(\xi \right)\right)}^{2y_{1}-1}\;{\left(\sin\left(\xi \right)\right)}^{2y_{2}-1}\;\psi_{\varrho ,\eta }^{\tau ,\mu }\left(\frac{-\delta }{{\left(\cos\left(\xi \right)\right)}^{2}{\left(\sin\left(\xi \right)\right)}^{2}}\right)d\xi .$$

*Proof. From (24), we yield*

$$B_{\delta ,\varrho ,\eta }^{\tau ,\mu }\left(\xi_{1},\;\xi_{2}\right)=\int_{0}^{1}{℘}^{\xi_{1}-1}{\left(1-℘\right)}^{\xi_{2}-1}\;\psi_{\varrho ,\eta }^{\tau ,\mu }\left(-\delta {\left(℘\left(1-℘\right)\right)}^{-1}\right)\;d℘$$

*Putting* $℘={\left(\cos(\xi )\right)}^{2}$ *and* d℘=−2cos(ξ)sin(ξ)dξ *in the above equation, we attain*

$$B_{\delta ,\varrho ,\eta }^{\tau ,\mu }\;\left(\xi_{1},\;\xi_{2}\right)=2\int_{0}^{\frac{\pi }{2}}{\left(\cos\left(\xi \right)\right)}^{2y_{1}-1}\;{\left(\sin\left(\xi \right)\right)}^{2y_{2}-1}\;\psi_{\varrho ,\eta }^{\tau ,\mu }\left(\frac{-\delta }{{\left(\cos\left(\xi \right)\right)}^{2}{\left(\sin\left(\xi \right)\right)}^{2}}\right)d\xi .$$

Theorem 8 *For*δ≥0*, and*$\min\{R\left(\xi_{1}\right),\;R\left(\xi_{2}\right),\;R\left(\tau \right),\;R\left(\mu \right),\;R\left(\varrho \right),\;R\left(\eta \right)\}>0$*, then the extended Beta function*$B_{\delta ,\varrho ,\eta }^{\tau ,\mu }\left(\xi_{1},\;\xi_{2}\right)$*in (24) achieves the following relation*.

(32) $$B_{\delta ,\varrho ,\eta }^{\tau ,\mu }\left(\xi_{1},\;\xi_{2}\right)=\sum_{\kappa =0}^{n}\frac{n!}{\kappa !\left(n-\kappa \right)!}\;B_{\delta ,\varrho ,\eta }^{\tau ,\mu }\left(\xi_{1}+\kappa ,\;\xi_{2}+n-\kappa \right).$$

*Proof. From* Eq. (26) *, we attain*

$$B_{\delta ,\varrho ,\eta }^{\tau ,\mu }\left(\xi_{1},\;\xi_{2}\right)=B_{\delta ,\varrho ,\eta }^{\tau ,\mu }\left(\xi_{1},\;\xi_{2}+1\right)+B_{\delta ,\varrho ,\eta }^{\tau ,\mu }\left(\xi_{1}+1,\;\xi_{2}\right).$$

*By employing (26) in the above equation, we acquire*

$$B_{\delta ,\varrho ,\eta }^{\tau ,\mu }\left(\xi_{1},\;\xi_{2}\right)=B_{\delta ,\varrho ,\eta }^{\tau ,\mu }\left(\xi_{1}+2,\;\xi_{2}\right)+2B_{\delta ,\varrho ,\eta }^{\tau ,\mu }\left(\xi_{1}+1,\;\xi_{2}+1\right)+B_{\delta ,\varrho ,\eta }^{\tau ,\mu }\left(\xi_{1},\;\xi_{2}+2\right)$$

*Therefore,*

$$B_{\delta ,\varrho ,\eta }^{\tau ,\mu }\left(\xi_{1},\;\xi_{2}\right)=\sum_{\kappa =0}^{n}\frac{n!}{\kappa !\left(n-\kappa \right)!}\;B_{\delta ,\varrho ,\eta }^{\tau ,\mu }\left(\xi_{1}+\kappa ,\;\xi_{2}+n-\kappa \right).$$

**Theorem 9.** For $m\in N_{0}$, $n\in N_{0}$, δ≥0, $\min\{R\left(\xi_{1}\right),\;R\left(\xi_{2}\right),\;R\left(\tau \right),\;R\left(\mu \right),\;R\left(\varrho \right),\;R\left(\eta \right)\}>0$, then the derivative formulas of the extended Beta function $B_{\delta ,\varrho ,\eta }^{\tau ,\mu }\left(\xi_{1},\;\xi_{2}\right)$ in (24) are provided by

(33) $$\frac{\partial^{m}}{\partial \xi_{1}^{m}}B_{\delta ,\varrho ,\eta }^{\tau ,\mu }\left(\xi_{1},\;\xi_{2}\right)=\int_{0}^{1}{℘}^{\xi_{1}-1}\ln^{m}\left(℘\right)\;{\left(1-℘\right)}^{\xi_{2}-1}\;\psi_{\varrho ,\eta }^{\tau ,\mu }\left(-\delta {\left(℘\left(1-℘\right)\right)}^{-1}\right)d℘,$$

(34) $$\frac{\partial^{n}}{\partial \xi_{2}^{n}}B_{\delta ,\varrho ,\eta }^{\tau ,\mu }\left(\xi_{1},\;\xi_{2}\right)=\int_{0}^{1}{℘}^{\xi_{1}-1}\;{\left(1-℘\right)}^{\xi_{2}-1}\;ln^{n}\left(1-℘\right)\;\psi_{\varrho ,\eta }^{\tau ,\mu }\left(-\delta {\left(℘\left(1-℘\right)\right)}^{-1}\right)d℘,$$

*and*

(35) $$\frac{\partial^{m+n}}{\partial \xi_{1}^{m}\partial \xi_{2}^{n}}B_{\delta ,\varrho ,\eta }^{\tau ,\mu }\left(\xi_{1},\;\xi_{2}\right)=\int_{0}^{1}{℘}^{\xi_{1}-1}\ln^{m}\left(℘\right)\;{\left(1-℘\right)}^{\xi_{2}-1}\;ln^{n}\left(1-℘\right)\;\psi_{\varrho ,\eta }^{\tau ,\mu }\left(-\delta {\left(℘\left(1-℘\right)\right)}^{-1}\right)d℘.$$

*Proof. By differentiating* m *-time for (24) with respect to* $\xi_{1},\;$ *we gain*

$$\frac{\partial^{m}}{\partial \xi_{1}^{m}}B_{\delta ,\varrho ,\eta }^{\tau ,\mu }\left(\xi_{1},\;\xi_{2}\right)=\frac{\partial^{m}}{\partial \xi_{1}^{m}}\int_{0}^{1}{℘}^{\xi_{1}-1}{\left(1-℘\right)}^{\xi_{2}-1}\;\psi_{\varrho ,\eta }^{\tau ,\mu }\left(-\delta {\left(℘\left(1-℘\right)\right)}^{-1}\right)\;d℘$$

$$=\int_{0}^{1}\left(\frac{\partial^{m}}{\partial \xi_{1}^{m}}\;{℘}^{\xi_{1}}\right){℘}^{-1}{\left(1-℘\right)}^{\xi_{2}-1}\;\psi_{\varrho ,\eta }^{\tau ,\mu }\left(-\delta {\left(℘\left(1-℘\right)\right)}^{-1}\right)\;d℘.$$

*We also notice that* $\frac{\partial^{m}}{\partial \xi_{1}^{m}}\;{℘}^{\xi_{1}}={℘}^{\xi_{1}}\ln^{m}\left(℘\right)$ *, hence*

$$\frac{\partial^{m}}{\partial \xi_{1}^{m}}B_{\delta ,\varrho ,\eta }^{\tau ,\mu }\left(\xi_{1},\;\xi_{2}\right)=\int_{0}^{1}{℘}^{\xi_{1}-1}\ln^{m}\left(℘\right)\;{\left(1-℘\right)}^{\xi_{2}-1}\;\psi_{\varrho ,\eta }^{\tau ,\mu }\left(-\delta {\left(℘\left(1-℘\right)\right)}^{-1}\right)d℘\;.$$

*By analogous technique, we gain*

$$\frac{\partial^{n}}{\partial \xi_{2}^{n}}B_{\delta ,\varrho ,\eta }^{\tau ,\mu }\left(\xi_{1},\;\xi_{2}\right)=\frac{\partial^{n}}{\partial \xi_{2}^{n}}\int_{0}^{1}{℘}^{\xi_{1}-1}\;{\left(1-℘\right)}^{\xi_{2}-1}\;\psi_{\varrho ,\eta }^{\tau ,\mu }\left(-\delta {\left(℘\left(1-℘\right)\right)}^{-1}\right)d℘$$

$$=\int_{0}^{1}{℘}^{\xi_{1}-1}\;\left(\frac{\partial^{n}}{\partial \xi_{2}^{n}}\;{\left(1-℘\right)}^{\xi_{2}}\right)\;\;{\left(1-℘\right)}^{-1}\;\psi_{\varrho ,\eta }^{\tau ,\mu }\left(-\delta {\left(℘\left(1-℘\right)\right)}^{-1}\right)\;d℘$$

*Obviously,* $\frac{\partial^{n}}{\partial \xi_{2}^{n}}\;{\left(1-℘\right)}^{\xi_{2}}=\;{\left(1-℘\right)}^{\xi_{2}}\;ln^{n}\left(1-℘\right),$ *then*

$$\frac{\partial^{n}}{\partial \xi_{2}^{n}}B_{\delta ,\varrho ,\eta }^{\tau ,\mu }\left(\xi_{1},\;\xi_{2}\right)=\int_{0}^{1}{℘}^{\xi_{1}-1}\;{\left(1-℘\right)}^{\xi_{2}-1}\;ln^{n}\left(1-℘\right)\;\psi_{\varrho ,\eta }^{\tau ,\mu }\left(-\delta {\left(℘\left(1-℘\right)\right)}^{-1}\right)d℘.$$

*In the same manner, we produce the last part.*

**Theorem 10.** *For* δ≥0 *, and* $\min\{R\left(\xi_{1}\right),\;R\left(\xi_{2}\right),\;R\left(\tau \right),\;R\left(\mu \right),\;R\left(\varrho \right),\;R\left(\eta \right)\}>0$ *, then the derivative formulas for the extended Beta function* $B_{\delta ,\varrho ,\eta }^{\tau ,\mu }\left(\xi_{1},\;\xi_{2}\right)\;$ *in (23) as:*

$$\frac{\partial }{\partial \delta }B_{\delta ,\varrho ,\eta }^{\tau ,\mu }\left(\xi_{1},\;\xi_{2}\right)=\frac{1}{\;\delta \;}\int_{0}^{1}{℘}^{\xi_{1}-1}{\left(1-℘\right)}^{\xi_{2}-1}\;\sum_{\kappa =0}^{\infty }\;\frac{{\left(\tau \right)}_{\kappa }\;{\left(-\delta \right)}^{\kappa }}{\;\;{\left(\varrho \right)}_{\kappa }\left(\kappa -1\right)!\;{\left(\kappa +\eta \right)}^{\mu }\;{℘}^{\kappa }\;{\left(1-℘\right)}^{\kappa }}\;d℘.$$

*Proof. By differentiating for (24) with respect to* δ, *we attain:*

$$\frac{\partial }{\partial \delta }B_{\delta ,\varrho ,\eta }^{\tau ,\mu }\left(\xi_{1},\;\xi_{2}\right)=\frac{\partial }{\partial \delta }\int_{0}^{1}{℘}^{\xi_{1}-1}{\left(1-℘\right)}^{\xi_{2}-1}\;\sum_{\kappa =0}^{\infty }\frac{{\left(\tau \right)}_{\kappa }\;{\left(-\delta \right)}^{\kappa }}{\;\;{\left(\varrho \right)}_{\kappa }\left(\kappa \right)!\;{\left(\kappa +\eta \right)}^{\mu }\;{℘}^{\kappa }\;{\left(1-℘\right)}^{\kappa }}\;d℘$$

$$=\int_{0}^{1}{℘}^{\xi_{1}-1}{\left(1-℘\right)}^{\xi_{2}-1}\;\left(\frac{\partial }{\partial \delta }\right)\;\sum_{\kappa =0}^{\infty }\frac{\;{\left(\tau \right)}_{\kappa }}{\;{\left(\varrho \right)}_{\kappa }\;}\;\frac{{\left(-\delta \right)}^{\kappa }}{\;\kappa !\;{\left(\kappa +\eta \right)}^{\mu }\;{℘}^{\kappa }\;{\left(1-℘\right)}^{\kappa }}\;d℘$$

*Note that* $\frac{\partial }{\partial \delta }\;{\left(-\delta \right)}^{\kappa }=\frac{\;\kappa \;}{\;\delta \;}{\left(-\delta \right)}^{\kappa }$ *, thus*

$$\frac{\partial }{\partial \delta }B_{\delta ,\varrho ,\eta }^{\tau ,\mu }\left(\xi_{1},\;\xi_{2}\right)=\frac{1}{\;\delta \;}\int_{0}^{1}{℘}^{\xi_{1}-1}{\left(1-℘\right)}^{\xi_{2}-1}\;\sum_{\kappa =0}^{\infty }\frac{\;{\left(\tau \right)}_{\kappa }}{\;{\left(\varrho \right)}_{\kappa }\;}\;\frac{{\left(-\delta \right)}^{\kappa }}{\;\left(\kappa -1\right)!\;{\left(\kappa +\eta \right)}^{\mu }\;{℘}^{\kappa }\;{\left(1-℘\right)}^{\kappa }}\;d℘.$$

### Statistical distribution of $B_{\delta ,\varrho ,\eta }^{\tau ,\mu }\left(\xi_{1},\;\xi_{2}\right)$

This section deals with the implementation of Statistical Distribution Theory (SDT). The Beta distribution is extended by utilizing the extended Beta function mentioned in (24). The new extended Beta distribution is used, and the study revolves around its mean, variance, moment-generating function, and cumulative distribution function.

**Definition 2.** The extended Beta distribution, including the extended Beta function $B_{\delta ,\varrho ,\eta }^{\tau ,\mu }\left(\xi_{1},\;\xi_{2}\right)$ (24) is formulated by

(36) $$\Lambda \left(℘\right)=\{\begin{array}{c} \begin{array}{c} \frac{1}{B_{\delta ,\varrho ,\eta }^{\tau ,\mu }\left(\xi_{1},\;\xi_{2}\right)}{℘}^{\xi_{1}-1}{\left(1-℘\right)}^{\xi_{2}-1}\;\psi_{\varrho ,\eta }^{\tau ,\mu }\left(-\delta {\left(℘\left(1-℘\right)\right)}^{-1}\right),\;\left(0<℘<1\right)\\ \; \end{array}\\ 0,\;otherwise\; \end{array}\;$$

The extended Beta distribution with parameters $-\infty <\xi_{1},\;\xi_{2}<\infty ,$ δ≥0and min{R(τ),R(μ),R(ϱ),R(η)}>0, will be used on a random variable χ with a probability density function (pdf) stated by Λ(℘) (36).

For η∈R, we have

$$\Xi \left(\chi^{\lambda }\right)=\frac{\;B_{\delta ,\varrho ,\eta }^{\tau ,\mu }\left(\xi_{1}+\lambda ,\;\xi_{2}\right)\;}{B_{\delta ,\varrho ,\eta }^{\tau ,\mu }\left(\xi_{1},\;\xi_{2}\right)},$$

Particularly, for λ=1, the mean of the distribution is

$$Mean=\Xi \left(\chi \right)=\frac{B_{\delta ,\varrho ,\eta }^{\tau ,\mu }\left(\xi_{1}+1,\;\xi_{2}\right)}{B_{\delta ,\varrho ,\eta }^{\tau ,\mu }\left(\xi_{1},\;\xi_{2}\right)}.$$

Therefore, the variance of the distribution is

$$Var\left(\chi \right)=E\left(\chi^{2}\right)-{\left\{E\left(\chi \right)\right\}}^{2}=\frac{B_{\delta ,\varrho ,\eta }^{\tau ,\mu }\left(\xi_{1},\;\xi_{2}\right)\;B_{\delta ,\varrho ,\eta }^{\tau ,\mu }\left(\xi_{1}+2,\;\xi_{2}\right)-{\left[B_{\delta ,\varrho ,\eta }^{\tau ,\mu }\left(\xi_{1}+1,\;\xi_{2}\right)\right]}^{2}}{{\left[B_{\delta ,\varrho ,\eta }^{\tau ,\mu }\left(\xi_{1},\;\xi_{2}\right)\right]}^{2}}.$$

The moment generation function (mgf) of the distribution is

$$m_{\chi }\left(m\right)=\sum_{\kappa =0}^{\infty }\frac{m^{\kappa }}{\kappa !}E\left(\chi^{\kappa }\right)=\frac{1}{B_{\delta ,\varrho ,\eta }^{\tau ,\mu }\left(\xi_{1},\;\xi_{2}\right)}\sum_{\kappa =0}^{\infty }\frac{m^{\kappa }}{\kappa !}B_{\delta ,\varrho ,\eta }^{\tau ,\mu }\left(\xi_{1}+\kappa ,\;\xi_{2}\right).$$

The cumulative distribution of Λ(℘) (36) is considered as:

$$\varphi \left(\varkappa \right)=\frac{B_{\delta ,\varrho ,\eta ,\varkappa }^{\tau ,\mu }\left(\xi_{1},\;\xi_{2}\right)}{B_{\delta ,\varrho ,\eta }^{\tau ,\mu }\left(\xi_{1},\;\xi_{2}\right)},$$

where

(37) $$B_{\delta ,\varrho ,\eta ,\varkappa }^{\tau ,\mu }\left(\xi_{1},\;\xi_{2}\right)=\int_{0}^{\varkappa }{℘}^{\xi_{1}-1}{\left(1-℘\right)}^{\xi_{2}-1}\;\psi_{\varrho ,\eta }^{\tau ,\mu }\left(-\delta {\left(℘\left(1-℘\right)\right)}^{-1}\right)d℘,$$

$$\left(-\infty <\xi_{1},\;\xi_{2}<\infty ,\delta \geq 0,\;\min\left\{R\left(\tau \right),\;R\left(\mu \right),\;R\left(\varrho \right),\;R\left(\eta \right)\right\}>0\right)$$

is the new extended incomplete Beta function. For δ=μ=0, τ=ϱ=1, $\xi_{1},\;\xi_{2}>0$, Eq. (37) converges and $B_{0,1,\eta ,\varkappa }^{1,0}\left(\xi_{1},\;\xi_{2}\right)=B_{\varkappa }\left(\xi_{1},\;\xi_{2}\right)$, where $B_{\varkappa }\left(\xi_{1},\;\xi_{2}\right)$ is the incomplete Beta function [54] given as:

$$B_{\varkappa }\left(\xi_{1},\;\xi_{2}\right)=B_{0,1,\eta ,\varkappa }^{1,0}\left(\xi_{1},\;\xi_{2}\right)=\varkappa^{\xi_{1}}\sum_{\kappa =0}^{\infty }\frac{{\left(1-\xi_{2}\right)}_{\kappa }}{\kappa !\;\left(\varkappa +\kappa \right)}\varkappa^{\kappa }=\frac{\varkappa^{\xi_{1}}}{\xi_{1}}\;{\;}_{2}F_{1}\left(\xi_{1},\;1-\xi_{2};\xi_{1}+1;\varkappa \right).$$

This leads to the observation that the problem of formulating $B_{\delta ,\varrho ,\eta ,\varkappa }^{\tau ,\mu }\left(\xi_{1},\;\xi_{2}\right)$ related to certain special functions stays open. Possibly, this distribution should be advantageous in extending the statistical outcomes for variables that are strictly positive to address those variables which can arbitrarily have large negative values.

## Extended ordinary hypergeometric and kummer functions

This section introduces and analyzes the new extended ordinary hypergeometric function and Kummer function, which are related to $B_{\delta ,\varrho ,\eta }^{\tau ,\mu }\left(\xi_{1},\;\xi_{2}\right)$ in (24).

**Definition 3.** The extended ordinary hypergeometric function is formulated via the extended Beta function (24) and formulated as:

(38) $$F_{\delta ,\varrho ,\eta }^{\tau ,\mu }\left(\xi_{1},\;\xi_{2};\xi_{3};z\right)=\sum_{\kappa =0}^{\infty }{\left(\xi_{1}\right)}_{\kappa }\frac{B_{\delta ,\varrho ,\eta }^{\tau ,\mu }\left(\xi_{2}+\kappa ,\xi_{3}-\xi_{2}\right)}{B\left(\xi_{2},\xi_{3}-\xi_{2}\right)}\;\frac{\;z^{\kappa }}{\kappa !},\;$$

where $\min\{R\left(\xi_{1}\right),\;R\left(\xi_{2}\right),\;R\left(\xi_{3}\right)\}>0$ and |z|<1.

Definition 4.2. The extended Kummer function correlated with the extended Beta function (24) is coined as:

(39) $$K_{\delta ,\varrho ,\eta }^{\tau ,\mu }\left(\xi_{2};\xi_{3};z\right)=\sum_{\kappa =0}^{\infty }\frac{B_{\delta ,\varrho ,\eta }^{\tau ,\mu }\left(\xi_{2}+\kappa ,\xi_{3}-\;\xi_{2}\right)}{B\left(\xi_{2},\xi_{3}-\xi_{2}\right)}\;\frac{\;z^{\kappa }}{\kappa !},\;$$

where $\min\{\;R\left(\xi_{1}\right),\;R\left(\xi_{2}\right)\}>0$ and |z|<1.

Now, the integral representations of the extended ordinary hypergeometric function (38) and Kummer function (39) will be investigated.

**Theorem 11** *. For* $\xi_{1},\;\xi_{2},\;\xi_{3}\in C$ *and* $\min\{R\left(\xi_{1}\right),\;R\left(\xi_{2}\right),\;R\left(\xi_{3}\right)\}>0$ *, then the extended ordinary hypergeometric function* $F_{\delta ,\varrho ,\eta }^{\tau ,\mu }\left(\xi_{1},\;\xi_{2};\xi_{3};z\right)$ *in (38) has the following integral representation:*

(40) $$F_{\delta ,\varrho ,\eta }^{\tau ,\mu }\left(\xi_{1},\;\xi_{2};\xi_{3};z\right)=\frac{1}{B\left(\xi_{2},\xi_{3}-\xi_{2}\right)}\int_{0}^{1}{℘}^{\xi_{2}-1}\;{\left(1-℘\right)}^{\xi_{3}-\xi_{2}-1}\;{\left(1-℘z\right)}^{-\xi_{1}}\;\times \psi_{\varrho ,\eta }^{\tau ,\mu }\left(-\delta {\left(℘\left(1-℘\right)\right)}^{-1}\right)d℘.$$

*Proof. From (38), (24) and binomial expansion* ${\left(1-℘\right)}^{-\xi_{2}}=\sum_{\kappa =0}^{\infty }\frac{{\left(\xi_{2}\right)}_{\kappa }}{\;\kappa !\;}{℘}^{\kappa }$ *,* |℘|<1 *, we gain*

$$F_{\delta ,\varrho ,\eta }^{\tau ,\mu }\left(\xi_{1},\;\xi_{2};\xi_{3};z\right)=\sum_{\kappa =0}^{\infty }\begin{array}{c} {\left(\xi_{1}\right)}_{\kappa }\frac{B_{\delta ,\varrho ,\eta }^{\tau ,\mu }\left(\xi_{2}+\kappa ,\xi_{3}-\xi_{2}\right)}{B\left(\xi_{2},\xi_{3}-\xi_{2}\right)}\;\frac{\;z^{\kappa }}{\kappa !}\\ \; \end{array}$$

$$=\frac{1}{B\left(\xi_{2},\xi_{3}-\xi_{2}\right)}\int_{0}^{1}{℘}^{\xi_{2}-1}\;{\left(1-℘\right)}^{\xi_{3}-\xi_{2}-1}\;\sum_{\kappa =0}^{\infty }\begin{array}{c} {\left(\xi_{1}\right)}_{\kappa }\;\frac{\;{\left(℘z\right)}^{\kappa }}{\kappa !}\\ \; \end{array}\;\psi_{\varrho ,\eta }^{\tau ,\mu }\left(-\delta {\left(℘\left(1-℘\right)\right)}^{-1}\right)\;d℘$$

$$=\frac{1}{B\left(\xi_{2},\xi_{3}-\xi_{2}\right)}\int_{0}^{1}{℘}^{\xi_{2}-1}\;{\left(1-℘\right)}^{\xi_{3}-\xi_{2}-1}\;{\left(1-℘z\right)}^{-\xi_{1}}\;\psi_{\varrho ,\eta }^{\tau ,\mu }\left(-\delta {\left(℘\left(1-℘\right)\right)}^{-1}\right)\;d℘.$$

**Theorem 12.** *For* $\xi_{1},\;\xi_{2},\;\xi_{3}\in C$ *and* $\min\{R\left(\xi_{1}\right),\;R\left(\xi_{2}\right),\;R\left(\xi_{3}\right)\}>0$ *, then the integral representations for the extended ordinary hypergeometric function* $F_{\delta ,\varrho ,\eta }^{\tau ,\mu }\left(\xi_{1},\;\xi_{2};\xi_{3};z\right)$ *in (38) are provided by*

$$1.\;\;F_{\delta ,\varrho ,\eta }^{\tau ,\mu }\left(\xi_{1},\;\xi_{2};\xi_{3};z\right)=\frac{1}{B\left(\xi_{2},\xi_{3}-\xi_{2}\right)}\int_{0}^{1}\omega^{\xi_{2}-1}\;{\left(1+\omega \right)}^{\xi_{1}-\xi_{3}}\;{\left(1+\omega \left(1-z\right)\right)}^{-\xi_{1}}\;$$

(41) $$\times \psi_{\varrho ,\eta }^{\tau ,\mu }\left(\frac{-\delta {\left(1+\omega \right)}^{2}}{\omega }\right)d℘.$$

$$2.\;\;F_{\delta ,\varrho ,\eta }^{\tau ,\mu }\left(\xi_{1},\;\xi_{2};\xi_{3};z\right)=\frac{2}{B\left(\xi_{2},\xi_{3}-\xi_{2}\right)}\int_{0}^{1}{\left(sin\phi \right)}^{2\xi_{2}-1}\;{\left(cos\phi \right)}^{2\xi_{3}-2\xi_{2}-1}\;{\left(1-\omega \;sin^{2}\phi \right)}^{-\xi_{1}}\;$$

(42) $$\times \psi_{\varrho ,\eta }^{\tau ,\mu }\left(\frac{-\delta }{\;sin^{2}\phi \;\;cos^{2}\phi }\right)d\phi .$$

*Proof. By considering* $℘=\frac{\omega }{1+\omega }$ *and* $℘=\;sin^{2}\phi \;$ *in* Eq. (40) *, the outcomes are attained.*

**Theorem 13.** *For* $\xi_{1},\;\xi_{2}\in C$ *and* $\min\{R\left(\xi_{1}\right),\;R\left(\xi_{2}\right)\}>0$ *, then the extended Kummer function* $K_{\delta ,\varrho ,\eta }^{\tau ,\mu }\left(\xi_{2};\xi_{3};z\right)$ *in (39) has the following integral representation:*

(43) $$K_{\delta ,\varrho ,\eta }^{\tau ,\mu }\left(\xi_{2};\xi_{3};z\right)=\frac{1}{B\left(\xi_{2},\xi_{3}-\xi_{2}\right)}\int_{0}^{1}{℘}^{\xi_{2}-1}\;{\left(1-℘\right)}^{\xi_{3}-\xi_{2}-1}\;e^{℘z}\;\psi_{\varrho ,\eta }^{\tau ,\mu }\left(-\delta {\left(℘\left(1-℘\right)\right)}^{-1}\right)\;d℘.$$

*Proof. From (39), (24) and binomial expansion* $exp\left(℘\right)=\sum_{\kappa =0}^{\infty }\frac{{℘}^{\kappa }}{\;\kappa !\;}$ *, we acquire*

$$K_{\delta ,\varrho ,\eta }^{\tau ,\mu }\left(\xi_{2};\xi_{3};z\right)=\sum_{\kappa =0}^{\infty }\frac{B_{\delta ,\varrho ,\eta }^{\tau ,\mu }\left(\xi_{2}+\kappa ,\xi_{3}-\;\xi_{2}\right)}{B\left(\xi_{2},\xi_{3}-\xi_{2}\right)}\;\frac{\;z^{\kappa }}{\kappa !}\;$$

$$=\frac{1}{B\left(\xi_{2},\xi_{3}-\xi_{2}\right)}\int_{0}^{1}{℘}^{\xi_{2}-1}\;{\left(1-℘\right)}^{\xi_{3}-\xi_{2}-1}\;\sum_{\kappa =0}^{\infty }\begin{array}{c} \;\frac{\;{\left(℘z\right)}^{\kappa }}{\kappa !}\\ \; \end{array}\;\psi_{\varrho ,\eta }^{\tau ,\mu }\left(-\delta {\left(℘\left(1-℘\right)\right)}^{-1}\right)\;d℘$$

$$=\frac{1}{B\left(\xi_{2},\xi_{3}-\xi_{2}\right)}\int_{0}^{1}{℘}^{\xi_{2}-1}\;{\left(1-℘\right)}^{\xi_{3}-\xi_{2}-1}\;e^{℘z}\;\psi_{\varrho ,\eta }^{\tau ,\mu }\left(-\delta {\left(℘\left(1-℘\right)\right)}^{-1}\right)\;d℘.$$

**Theorem 14** *. For* $\xi_{1},\;\xi_{2}\in C$ *and* $\min\{R\left(\xi_{1}\right),\;R\left(\xi_{2}\right),\}>0$ *, then the integral representations for the extended Kummer function* $K_{\delta ,\varrho ,\eta }^{\tau ,\mu }\left(\xi_{2};\xi_{3};z\right)$ *in (39) hold:*

(44) $$1.\;K_{\delta ,\varrho ,\eta }^{\tau ,\mu }\left(\xi_{2};\xi_{3};z\right)=\frac{1}{B\left(\xi_{2},\xi_{3}-\xi_{2}\right)}\int_{0}^{1}\omega^{\xi_{2}-1}\;{\left(1+\omega \right)}^{\xi_{1}-\xi_{3}}\;exp\left(\frac{℘\omega }{1+\omega }\right)\;\times \psi_{\varrho ,\eta }^{\tau ,\mu }\left(\frac{-\delta {\left(1+\omega \right)}^{2}}{\omega }\right)d\phi .\;$$

(45) $$2.\;K_{\delta ,\varrho ,\eta }^{\tau ,\mu }\left(\xi_{2};\xi_{3};z\right)=\frac{2}{B\left(\xi_{2},\xi_{3}-\xi_{2}\right)}\int_{0}^{1}{\left(sin\phi \right)}^{2\xi_{2}-1}\;{\left(co\phi \right)}^{2\xi_{3}-2\xi_{2}-1}exp\left(\;cos^{2}\phi \right)\;\times \;\psi_{\varrho ,\eta }^{\tau ,\mu }\left(\frac{-\delta }{\;sin^{2}\phi \;\;cos^{2}\phi }\right)d\phi .$$

*Proof. By setting* $℘=\frac{\omega }{1+\omega }$ *and* $℘=\;sin^{2}\phi \;$ *in* Eq. (43) *, the outcomes are yielded.*

**Theorem 15.** *For* $\xi_{1},\;\xi_{2},\;\xi_{3}\in C$ *and* $\min\{R\left(\xi_{1}\right),\;R\left(\xi_{2}\right),\;R\left(\xi_{3}\right)\}>0$ *, then the differentiation formulas of the extended ordinary hypergeometric function* $F_{\delta ,\varrho ,\eta }^{\tau ,\mu }\left(\xi_{1},\;\xi_{2};\xi_{3};z\right)$ *in (38) is provided by*

(46) $$\frac{d^{m}}{dz^{m}}\left\{F_{\delta ,\varrho ,\eta }^{\tau ,\mu }\left(\xi_{1},\;\xi_{2};\xi_{3};z\right)\right\}=\frac{{\left(\xi_{1}\right)}_{m}\;{\left(\xi_{2}\right)}_{m}}{{\left(\xi_{3}\right)}_{m}}F_{\delta ,\varrho ,\eta }^{\tau ,\mu }\left(\xi_{1}+m,\;\xi_{2}+m;\xi_{3}+m;z\right).$$

*Proof. Differentiating* Eq. (38) *, we acquire*

$$F_{\delta ,\varrho ,\eta }^{\tau ,\mu }\left(\xi_{1},\;\xi_{2};\xi_{3};z\right)=\sum_{\kappa =1}^{\infty }\begin{array}{c} {\left(\xi_{1}\right)}_{\kappa }\frac{B_{\delta ,\varrho ,\eta }^{\tau ,\mu }\left(\xi_{2}+\kappa ,\xi_{3}-\xi_{2}\right)}{B\left(\xi_{2},\xi_{3}-\xi_{2}\right)}\;\frac{\;z^{\kappa -1}}{\left(\kappa -1\right)!}\\ \; \end{array}\;.$$

*Replacing* κ *by* κ−1

(47) $$\frac{d}{dz}\left\{F_{\delta ,\varrho ,\eta }^{\tau ,\mu }\left(\xi_{1},\;\xi_{2};\xi_{3};z\right)\right\}=\sum_{\kappa =0}^{\infty }\begin{array}{c} {\left(\xi_{1}\right)}_{\kappa +1}\frac{B_{\delta ,\varrho ,\eta }^{\tau ,\mu }\left(\xi_{2}+\kappa +1,\xi_{3}-\xi_{2}\right)}{B\left(\xi_{2},\xi_{3}-\xi_{2}\right)}\;\frac{\;z^{\kappa }}{\kappa !}\\ \; \end{array}\;.\;$$

*Utilizing* $B\left(s,t-s\right)=\frac{t}{s}B\left(s+1,t-s\right)$ *in* Eq. (47) *, we gain*

$$\frac{d}{dz}\left\{F_{\delta ,\varrho ,\eta }^{\tau ,\mu }\left(\xi_{1},\;\xi_{2};\xi_{3};z\right)\right\}=\sum_{\kappa =0}^{\infty }\begin{array}{c} {\left(\xi_{1}\right)}_{\kappa +1}\left(\frac{\xi_{2}}{\xi_{3}}\right)\frac{B_{\delta ,\varrho ,\eta }^{\tau ,\mu }\left(\xi_{2}+\kappa +1,\xi_{3}-\xi_{2}\right)}{B\left(\xi_{2}+1,\xi_{3}-\xi_{2}\right)\;}\;\frac{\;z^{\kappa }}{\kappa !}\\ \; \end{array}$$

$$=\sum_{\kappa =0}^{\infty }\begin{array}{c} {\left(\xi_{1}\right)}_{\kappa }\left(\frac{\xi_{1}\xi_{2}}{\xi_{3}}\right)\frac{B_{\delta ,\varrho ,\eta }^{\tau ,\mu }\left(\xi_{2}+\kappa +1,\xi_{3}-\xi_{2}\right)}{B\left(\xi_{2}+1,\xi_{3}-\xi_{2}\right)\;}\;\frac{\;z^{\kappa }}{\kappa !}\\ \; \end{array}=\frac{\xi_{1}\xi_{2}}{\xi_{3}}\;F_{\delta ,\varrho ,\eta }^{\tau ,\mu }\left(\xi_{1}+1,\;\xi_{2}+1;\xi_{3}+1;z\right).$$

*Again, by differentiating* Eq. (38) *, we yield*

$$\frac{d^{2}}{dz^{2}}\left\{F_{\delta ,\varrho ,\eta }^{\tau ,\mu }\left(\xi_{1},\;\xi_{2};\xi_{3};z\right)\right\}=\frac{\xi_{1}\left(\xi_{1}+1\right)\xi_{2}\left(\xi_{2}+1\right)}{\xi_{3}\left(\xi_{3}+1\right)}\;F_{\delta ,\varrho ,\eta }^{\tau ,\mu }\left(\xi_{1}+2,\;\xi_{2}+2;\xi_{3}+2;z\right).$$

*By continuing this process,* m *-times, the outcome is derived.*

**Theorem 16.** *For* $\xi_{1},\;\xi_{2}\in C$ *and* $\min\{R\left(\xi_{1}\right),\;R\left(\xi_{2}\right)\}>0$ *, then the differentiation formulas of the extended Kummer function* $K_{\delta ,\varrho ,\eta }^{\tau ,\mu }\left(\xi_{2};\xi_{3};z\right)$ *in (39) is provided by*

(48) $$\frac{d^{m}}{dz^{m}}\left\{\;K_{\delta ,\varrho ,\eta }^{\tau ,\mu }\left(\xi_{2};\xi_{3};z\right)\right\}=\frac{\;{\left(\xi_{2}\right)}_{m}}{{\left(\xi_{3}\right)}_{m}}F_{\delta ,\varrho ,\eta }^{\tau ,\mu }\left(\;\xi_{2}+m;\xi_{3}+m;z\right).$$

*Proof. Proceeding analogously, as in Theorem 4.5, leads to the desired outcome.*

**Theorem 17.** *For* $\xi_{1},\;\xi_{2},\;\xi_{3}\in C$ *and* $\min\{R\left(\xi_{1}\right),\;R\left(\xi_{2}\right),\;R\left(\xi_{3}\right)\}>0$ *, then the transformation formula of the extended ordinary hypergeometric function* $F_{\delta ,\varrho ,\eta }^{\tau ,\mu }\left(\xi_{1},\;\xi_{2};\xi_{3};z\right)$ *(38) is provided by*

(49) $$F_{\delta ,\varrho ,\eta }^{\tau ,\mu }\left(\xi_{1},\;\xi_{2};\xi_{3};z\right)={\left(1-z\right)}^{-\xi_{1}}\;F_{\delta ,\varrho ,\eta }^{\tau ,\mu }\left(\xi_{1},\xi_{3}-\;\xi_{2};\xi_{3};\frac{z}{1-z}\right)$$

*Proof. From* Eq. (40) *, we gain*

$$F_{\delta ,\varrho ,\eta }^{\tau ,\mu }\left(\xi_{1},\;\xi_{2};\xi_{3};z\right)=\frac{1}{B\left(\xi_{2},\xi_{3}-\xi_{2}\right)}\int_{0}^{1}{℘}^{\xi_{2}-1}\;{\left(1-℘\right)}^{\xi_{3}-\xi_{2}-1}\;{\left(1-℘z\right)}^{-\xi_{1}}$$

$$\times \psi_{\varrho ,\eta }^{\tau ,\mu }\left(-\delta {\left(℘\left(1-℘\right)\right)}^{-1}\right)d℘.$$

*Replacing* ℘ *by* 1−℘ *and by* ${\left[1-z(1-℘)\right]}^{-\xi_{1}}=\;{\left(1-z\right)}^{-\xi_{1}}\;{\left(1-\frac{℘z}{1-z}\right)}^{-\xi_{1}}$ *leads to*

$$F_{\delta ,\varrho ,\eta }^{\tau ,\mu }\left(\xi_{1},\;\xi_{2};\xi_{3};z\right)=\frac{{\left(1-z\right)}^{-\xi_{1}}}{B\left(\xi_{2},\xi_{3}-\xi_{2}\right)}\int_{0}^{1}{\left(1-℘\right)}^{\xi_{2}-1}\;{℘}^{\xi_{3}-\xi_{2}-1}\;{\left(1-\frac{℘z}{1-z}\right)}^{-\xi_{1}}$$

$$\times \psi_{\varrho ,\eta }^{\tau ,\mu }\left(-\delta {\left(℘\left(1-℘\right)\right)}^{-1}\right)d℘$$

$$=\frac{{\left(1-z\right)}^{-\xi_{1}}}{B\left(\xi_{2},\xi_{3}-\xi_{2}\right)}\sum_{\kappa =0}^{\infty }{\left(\xi_{1}\right)}_{\kappa }\int_{0}^{1}\;{℘}^{\xi_{3}-\xi_{2}+\kappa -1}{\left(1-℘\right)}^{\xi_{2}-1}\psi_{\varrho ,\eta }^{\tau ,\mu }\left(-\delta {\left(℘\left(1-℘\right)\right)}^{-1}\right)d℘\;\frac{\;{\left(\frac{z}{1-z}\right)}^{\kappa }}{\kappa !}$$

$$={\left(1-z\right)}^{-\xi_{1}}\sum_{\kappa =0}^{\infty }{\left(\xi_{1}\right)}_{\kappa }\frac{B_{\delta ,\varrho ,\eta }^{\tau ,\mu }\left(\xi_{3}-\xi_{2}+\kappa ,\xi_{2}\right)}{B\left(\xi_{2},\xi_{3}-\xi_{2}\right)}\frac{\;{\left(\frac{z}{1-z}\right)}^{\kappa }}{\kappa !}\;={\left(1-z\right)}^{-\xi_{1}}\;F_{\delta ,\varrho ,\eta }^{\tau ,\mu }\left(\xi_{1},\;\xi_{3}-\xi_{2};\xi_{3};\frac{z}{1-z}\right).$$

**Theorem 18.** *For* $\xi_{1},\;\xi_{2}\in C$ *and* $\min\{R\left(\xi_{1}\right),\;R\left(\xi_{2}\right)\}>0$ *, then the transformation formula of the extended Kummer function* $K_{\delta ,\varrho ,\eta }^{\tau ,\mu }\left(\xi_{2};\xi_{3};z\right)$ *in (39) is provided by*

(50) $$K_{\delta ,\varrho ,\eta }^{\tau ,\mu }\left(\xi_{2};\xi_{3};z\right)\;=e^{z}\;F_{\delta ,\varrho ,\eta }^{\tau ,\mu }\left(\xi_{3}-\;\xi_{2};\xi_{3};-z\right)$$

*Proof. From* Eq. (43) *, we gain*

$$K_{\delta ,\varrho ,\eta }^{\tau ,\mu }\left(\xi_{2};\xi_{3};z\right)=\frac{1}{B\left(\xi_{2},\xi_{3}-\xi_{2}\right)}\int_{0}^{1}{℘}^{\xi_{2}-1}\;{\left(1-℘\right)}^{\xi_{3}-\xi_{2}-1}\;e^{℘z}\;\psi_{\varrho ,\eta }^{\tau ,\mu }\left(-\delta {\left(℘\left(1-℘\right)\right)}^{-1}\right)\;d℘.$$

*Replacing* ℘ *by* 1−℘ *and by* $e^{(1-℘)z}=e^{z}\;e^{-℘z}$ *leads to*

$$K_{\delta ,\varrho ,\eta }^{\tau ,\mu }\left(\xi_{2};\xi_{3};z\right)=\frac{e^{z}}{B\left(\xi_{2},\xi_{3}-\xi_{2}\right)}\int_{0}^{1}{\left(1-℘\right)}^{\xi_{2}-1}\;{℘}^{\xi_{3}-\xi_{2}-1}\;e^{-℘z}\;\psi_{\varrho ,\eta }^{\tau ,\mu }\left(-\delta {\left(℘\left(1-℘\right)\right)}^{-1}\right)d℘$$

$$K_{\delta ,\varrho ,\eta }^{\tau ,\mu }\left(\xi_{2};\xi_{3};z\right)=\frac{e^{z}}{B\left(\xi_{2},\xi_{3}-\xi_{2}\right)}\int_{0}^{1}\;{℘}^{\xi_{3}-\xi_{2}+\kappa -1}{\left(1-℘\right)}^{\xi_{2}-1}\;e^{-z}\psi_{\varrho ,\eta }^{\tau ,\mu }\left(-\delta {\left(℘\left(1-℘\right)\right)}^{-1}\right)d℘$$

$$=e^{z}\sum_{\kappa =0}^{\infty }\frac{B_{\delta ,\varrho ,\eta }^{\tau ,\mu }\left(\xi_{3}-\xi_{2}+\kappa ,\xi_{2}\right)}{B\left(\xi_{2},\xi_{3}-\xi_{2}\right)}\frac{\;z^{\kappa }}{\kappa !}=e^{z}\;K_{\delta ,\varrho ,\eta }^{\tau ,\mu }\left(\xi_{3}-\xi_{2};\xi_{3};z\right)$$

**Theorem 19.** *For* $\xi_{1},\;\xi_{2},\;\xi_{3}\in C$ *,* $\min\{R\left(\xi_{1}\right),\;R\left(\xi_{2}\right),\;R\left(\xi_{3}\right)\}>0$ *and* |℘|<1 *, then the generating function for* $F_{\delta ,\varrho ,\eta }^{\tau ,\mu }\left(\xi_{1},\;\xi_{2};\xi_{3};z\right)$ *in (38) is provided by*

(51) $$\sum_{m=0}^{\infty }{\left(\xi_{1}\right)}_{m}\;F_{\delta ,\varrho ,\eta }^{\tau ,\mu }\left(\xi_{1}+m,\;\xi_{2};\xi_{3};z\right)\frac{\;\omega^{m}}{m!}={\left(1-\omega \right)}^{-\xi_{1}}\;F_{\delta ,\varrho ,\eta }^{\tau ,\mu }\left(\xi_{1},\xi_{3}-\;\xi_{2};\xi_{3};\frac{z}{1-\omega }\right).$$

*Proof. Let* T *be the left side of* Eq. (51) *. From* Eq. (38) *, we acquire*

$$T=\sum_{m=0}^{\infty }{\left(\xi_{1}\right)}_{m}\left\{\sum_{\kappa =0}^{\infty }\begin{array}{c} {\left(\xi_{1}+m\right)}_{\kappa }\frac{B_{\delta ,\varrho ,\eta }^{\tau ,\mu }\left(\xi_{2}+\kappa ,\xi_{3}-\xi_{2}\right)}{B\left(\xi_{2},\xi_{3}-\xi_{2}\right)}\;\frac{\;z^{\kappa }}{\kappa !}\\ \; \end{array}\right\}\frac{\;\omega^{m}}{m!}.$$

*Utilizing* ${\left(\xi_{1}\right)}_{m}{\left(\xi_{1}+m\right)}_{\kappa }={\left(\xi_{1}\right)}_{\kappa }\;{\left(\xi_{1}+\kappa \right)}_{m}\;$ *leads to*

$$T=\sum_{\kappa =0}^{\infty }{\left(\xi_{1}\right)}_{\kappa }\;\frac{B_{\delta ,\varrho ,\eta }^{\tau ,\mu }\left(\xi_{2}+\kappa ,\xi_{3}-\xi_{2}\right)}{B\left(\xi_{2},\xi_{3}-\xi_{2}\right)}\;\left\{\sum_{m=0}^{\infty }\begin{array}{c} {\left(\xi_{1}+\kappa \right)}_{m}\frac{\;\omega^{m}}{m!}\\ \; \end{array}\right\}\frac{\;z^{\kappa }}{\kappa !}\;.$$

*From binomial expansion* ${\left(1-\omega \right)}^{-(\xi_{1}+\kappa )}=\sum_{\kappa =0}^{\infty }{\left(\xi_{1}+\kappa \right)}_{m}\frac{\;\omega^{m}}{m!}$ *,* |℘|<1 *, we yield*

$$T=\sum_{\kappa =0}^{\infty }{\left(\xi_{1}\right)}_{\kappa }\;\frac{B_{\delta ,\varrho ,\eta }^{\tau ,\mu }\left(\xi_{2}+\kappa ,\xi_{3}-\xi_{2}\right)}{B\left(\xi_{2},\xi_{3}-\xi_{2}\right)}\;{\left(1-\omega \right)}^{-\left(\xi_{1}+\kappa \right)}\frac{\;z^{\kappa }}{\kappa !}$$

$$={\left(1-\omega \right)}^{-\xi_{1}}\sum_{\kappa =0}^{\infty }{\left(\xi_{1}\right)}_{\kappa }\;\frac{B_{\delta ,\varrho ,\eta }^{\tau ,\mu }\left(\xi_{2}+\kappa ,\xi_{3}-\xi_{2}\right)}{B\left(\xi_{2},\xi_{3}-\xi_{2}\right)}\;\frac{\;{\left(\frac{z}{1-\omega }\right)}^{\kappa }}{\kappa !}={\left(1-\omega \right)}^{-\xi_{1}}\;F_{\delta ,\varrho ,\eta }^{\tau ,\mu }\left(\xi_{1},\xi_{3}-\;\xi_{2};\xi_{3};\frac{z}{1-\omega }\right).$$

**Theorem 20.** *For* $\xi_{1},\;\xi_{2},\;\xi_{3}\in C$ *,* $\min\{R\left(\xi_{1}\right),\;R\left(\xi_{2}\right),\;R\left(\xi_{3}\right)\}>0$ *and* |℘|<1 *, then the generating function for* $F_{\delta ,\varrho ,\eta }^{\tau ,\mu }\left(\xi_{1},\;\xi_{2};\xi_{3};z\right)$ *in (38) is provided by*

(52) $$\sum_{m=0}^{\infty }\left(\begin{array}{c} \xi_{1}+m-1\\ m \end{array}\right)F_{\delta ,\varrho ,\eta }^{\tau ,\mu }\left(\xi_{1}+m,\;\xi_{2};\xi_{3};z\right)\;\omega^{m}={\left(1-\omega \right)}^{-\xi_{1}}\;F_{\delta ,\varrho ,\eta }^{\tau ,\mu }\left(\xi_{1},\xi_{3}-\;\xi_{2};\xi_{3};\frac{z}{1-\omega }\right).$$

*Proof. Let* T *be the left side of* Eq. (52) *. From* Eq. (38) *, we acquire*

$$T=\sum_{m=0}^{\infty }\left(\begin{array}{c} \xi_{1}+m-1\\ m \end{array}\right)\left\{\sum_{\kappa =0}^{\infty }\begin{array}{c} {\left(\xi_{1}+m\right)}_{\kappa }\frac{B_{\delta ,\varrho ,\eta }^{\tau ,\mu }\left(\xi_{2}+\kappa ,\xi_{3}-\xi_{2}\right)}{B\left(\xi_{2},\xi_{3}-\xi_{2}\right)}\;\frac{\;z^{\kappa }}{\kappa !}\\ \; \end{array}\right\}\;\omega^{m}.$$

*Utilizing* $\left(\begin{array}{c} \xi_{1}\\ m \end{array}\right)=\frac{\Gamma (\xi_{1}+1)}{m!\;\Gamma (\xi_{1}-m+1)},\;\left(m\in N_{0},\;\xi_{1}\in C\right)$ *leads to*

$$\Omega =\sum_{\kappa =0}^{\infty }\;\frac{B_{\delta ,\varrho ,\eta }^{\tau ,\mu }\left(\xi_{2}+\kappa ,\xi_{3}-\xi_{2}\right)}{B\left(\xi_{2},\xi_{3}-\xi_{2}\right)}\;\left\{\sum_{m=0}^{\infty }\begin{array}{c} \left(\begin{array}{c} \xi_{1}+m-1\\ m \end{array}\right){\left(\xi_{1}+\kappa \right)}_{m}\;\omega^{m}\\ \; \end{array}\right\}\frac{\;z^{\kappa }}{\kappa !}$$

$$=\sum_{\kappa =0}^{\infty }\;\frac{B_{\delta ,\varrho ,\eta }^{\tau ,\mu }\left(\xi_{2}+\kappa ,\xi_{3}-\xi_{2}\right)}{B\left(\xi_{2},\xi_{3}-\xi_{2}\right)}\;\left\{\sum_{m=0}^{\infty }\begin{array}{c} \left(\begin{array}{c} \xi_{1}+\kappa +m-1\\ m \end{array}\right){\left(\xi_{1}\right)}_{\kappa }\;\omega^{m}\\ \; \end{array}\right\}\frac{\;z^{\kappa }}{\kappa !}$$

$$=\sum_{\kappa =0}^{\infty }{\left(\xi_{1}\right)}_{\kappa }\;\frac{B_{\delta ,\varrho ,\eta }^{\tau ,\mu }\left(\xi_{2}+\kappa ,\xi_{3}-\xi_{2}\right)}{B\left(\xi_{2},\xi_{3}-\xi_{2}\right)}\;\left\{\sum_{m=0}^{\infty }\left(\begin{array}{c} \xi_{1}+\kappa +m-1\\ m \end{array}\right)\;\omega^{m}\right\}\frac{\;z^{\kappa }}{\kappa !}$$

$$=\sum_{\kappa =0}^{\infty }{\left(\xi_{1}\right)}_{\kappa }\;\frac{B_{\delta ,\varrho ,\eta }^{\tau ,\mu }\left(\xi_{2}+\kappa ,\xi_{3}-\xi_{2}\right)}{B\left(\xi_{2},\xi_{3}-\xi_{2}\right)}\;{\left(1-\omega \right)}^{-\left(\xi_{1}+\kappa \right)}\frac{\;z^{\kappa }}{\kappa !}$$

$$={\left(1-\omega \right)}^{-\xi_{1}}\sum_{\kappa =0}^{\infty }{\left(\xi_{1}\right)}_{\kappa }\;\frac{B_{\delta ,\varrho ,\eta }^{\tau ,\mu }\left(\xi_{2}+\kappa ,\xi_{3}-\xi_{2}\right)}{B\left(\xi_{2},\xi_{3}-\xi_{2}\right)}\;\frac{\;{\left(\frac{z}{1-\omega }\right)}^{\kappa }}{\kappa !}={\left(1-\omega \right)}^{-\xi_{1}}\;F_{\delta ,\varrho ,\eta }^{\tau ,\mu }\left(\xi_{1},\xi_{3}-\;\xi_{2};\xi_{3};\frac{z}{1-\omega }\right).$$

### Extended Riemann-Liouville fractional type derivative operator

This section introduces the new extended Riemann-Liouville (RL) type fractional derivative operator, which stems from the considered special function expressed in (23).

**Definition 4.** The extended Riemann-Liouville fractional derivative operator (RL-fractional derivative operator) of Φ(z) of order q is demarcated as: for R(q)<0

(53) $$D_{z,\delta }^{q}\Phi \left(z\right)=\frac{1}{\Gamma \left(-q\right)}\int_{0}^{z}\Phi \left(℘\right){\left(z-℘\right)}^{-q-1}\;\psi_{\varrho ,\eta }^{\tau ,\mu }\left(\frac{-\delta z^{2}}{℘\left(z-℘\right)}\right)d℘,\;\left(0\leq \delta \;\right).$$

For l−1<R(q)<l, where l∈N, the RL-fractional derivative operator structure is formulated as follows:

(54) $$D_{z,\delta }^{q}\Phi \left(z\right)=\frac{d^{l}}{dz^{l}}D_{z,\delta }^{q-l}\left\{\Phi \left(z\right)\right\}=\frac{1}{\Gamma \left(l-q\right)}\int_{0}^{z}\Phi \left(℘\right){\left(z-℘\right)}^{l-q-1}\;\psi_{\varrho ,\eta }^{\tau ,\mu }\left(\frac{-\delta z^{2}}{℘\left(z-℘\right)}\right)d℘,\left(0\leq \delta \;\right).$$

Obviously, for τ=ϱ=1 and μ=δ=0, it reduces to the classical RL- fractional derivative operator. The following outcomes consider the extended RL- fractional derivative operator of the function $z^{ℷ}$.

***Lemma 1.*** *Considering* R(q)<0 *, and* −1<R(ℷ) *, we have*

$$D_{z,\delta }^{q}\left\{z^{ℷ}\right\}=\frac{\;B_{\delta ,\varrho ,\eta }^{\tau ,\mu }\left(ℷ+1,\;-q\right)}{\Gamma \left(-q\right)}\;z^{ℷ-q}\;.$$

*Proof. Utilizing (53) to* $z^{ℷ}$ *, it produces*

(55) $$D_{z,\delta }^{q}\left\{z^{ℷ}\right\}=\frac{1}{\Gamma \left(-q\right)}\int_{0}^{z}{℘}^{ℷ}\;{\left(z-℘\right)}^{-q-1}\;\psi_{\varrho ,\eta }^{\tau ,\mu }\left(\frac{-\delta z^{2}}{℘\left(z-℘\right)}\right)d℘\;.$$

*Setting* ℘=zν *in (55) reduces to*

$$D_{z,\delta }^{q}\left\{z^{ℷ}\right\}=\frac{z^{ℷ-q}}{\Gamma \left(-q\right)}\int_{0}^{1}\nu^{ℷ+1-1}\;{\left(1-\nu \right)}^{-q-1}\;\psi_{\varrho ,\eta }^{\tau ,\mu }\left(\frac{-\delta }{\nu \left(1-\nu \right)}\right)d\nu =\frac{\;B_{\delta ,\varrho ,\eta }^{\tau ,\mu }\left(ℷ+1,\;-q\right)}{\Gamma \left(-q\right)}\;z^{ℷ-q}\;.$$

***Lemma 2.*** *Considering* l−1<R(q)<l *,* l∈N *, and* R(q)<R(ℷ) *, we have*

$$D_{z,\delta }^{q}\left\{z^{ℷ}\right\}=\frac{\Gamma \left(ℷ+1\right)\;B_{\delta ,\varrho ,\eta }^{\tau ,\mu }\left(ℷ+1,\;l-q\right)}{\Gamma \left(ℷ-q+1\right)\;B\left(ℷ+1,\;l-q\right)}\;z^{ℷ-q}\;.$$

*Proof. Employing (54) to* $z^{ℷ}$ *, it yields*

(56) $$D_{z,\delta }^{q}\left\{z^{ℷ}\right\}=\frac{d^{l}}{dz^{l}}\left\{\frac{1}{\Gamma \left(l-q\right)}\int_{0}^{z}{℘}^{ℷ}\;{\left(z-℘\right)}^{l-q-1}\;\psi_{\varrho ,\eta }^{\tau ,\mu }\left(\frac{-\delta z^{2}}{℘\left(z-℘\right)}\right)d℘\right\}.$$

*Putting* ℘=zν *in (56) reduces to*

$$D_{z,\delta }^{q}\left\{z^{ℷ}\right\}=\left(\frac{d^{l}}{dz^{l}}\;z^{l+ℷ-q}\right)\frac{1}{\Gamma \left(l-q\right)}\int_{0}^{1}\nu^{ℷ+1-1}\;{\left(1-\nu \right)}^{l-q-1}\;\psi_{\varrho ,\eta }^{\tau ,\mu }\left(\frac{-\delta }{\nu \left(1-\nu \right)}\right)d\nu =\left(\frac{d^{l}}{dz^{l}}\;z^{l+ℷ-q}\right)\frac{B_{\delta ,\varrho ,\eta }^{\tau ,\mu }\left(ℷ+1,\;l-q\right)}{\Gamma \left(l-q\right)}\;.$$

*Considering*

$$\frac{d^{l}}{dz^{l}}\;z^{l+ℷ-q}=\frac{\Gamma \left(l+ℷ-q+1\right)}{\Gamma \left(ℷ-q+1\right)}z^{ℷ-q}\;.$$

*Thus*

$$D_{z,\delta }^{q}\left\{z^{ℷ}\right\}=\frac{\Gamma \left(l+ℷ-q+1\right)\;B_{\delta ,\varrho ,\eta }^{\tau ,\mu }\left(ℷ+1,\;l-q\right)}{\Gamma \left(l-q\right)\;\Gamma \left(ℷ-q+1\right)}\;z^{ℷ-q}=\frac{\Gamma \left(ℷ+1\right)\;B_{\delta ,\varrho ,\eta }^{\tau ,\mu }\left(ℷ+1,\;l-q\right)}{\Gamma \left(ℷ-q+1\right)\;B\left(ℷ+1,\;l-q\right)}\;z^{ℷ-q}\;.$$

*Next, we investigate the analytic function at the origin in the sense of the extended RL-fractional derivative operator.*

**Theorem 21.** *Let* R(q)<0 *and* Q(z) *be an analytic function with its expansion stated by* $Q\left(z\right)=\sum_{\kappa =0}^{\infty }a_{\kappa }\;z^{\kappa }$ *in* |z|<r (r>0) *, then*

(57) $$D_{z,\delta }^{q}\left\{Q\left(z\right)\right\}=\sum_{\kappa =0}^{\infty }a_{\kappa }\;D_{z,\delta }^{q}\;\left\{z^{\kappa }\right\}=\frac{\;z^{-q}}{\Gamma \left(-q\right)}\sum_{\kappa =0}^{\infty }a_{\kappa }\;B_{\delta ,\varrho ,\eta }^{\tau ,\mu }\left(\kappa +1,\;-q\right)\;z^{\kappa }\;.$$

*Proof. Using (53) to the function* Q(z) *, it leads*

(58) $$D_{z,\delta }^{q}\left\{Q\left(z\right)\right\}=\;D_{z,\delta }^{q}\left\{\sum_{\kappa =0}^{\infty }a_{\kappa }\;z^{\kappa }\right\}=\frac{1}{\Gamma \left(-q\right)}\int_{0}^{z}\sum_{\kappa =0}^{\infty }a_{\kappa }\;{℘}^{\kappa }{\left(z-℘\right)}^{-q-1}\;\psi_{\varrho ,\eta }^{\tau ,\mu }\left(\frac{-\delta z^{2}}{℘\left(z-℘\right)}\right)d℘\;.$$

*Since the series is uniformly convergent in* |z|<r *and the integral is convergent, the order of summation and integration can be interchangeable. Hence, Eq. (58) reduces to*

$$D_{z,\delta }^{q}\left\{Q\left(z\right)\right\}=\sum_{\kappa =0}^{\infty }a_{\kappa }\left\{\frac{1}{\Gamma \left(-q\right)}\int_{0}^{z}{℘}^{\kappa }\;{\left(z-℘\right)}^{-q-1}\;\psi_{\varrho ,\eta }^{\tau ,\mu }\left(\frac{-\delta z^{2}}{℘\left(z-℘\right)}\right)\right\}=\sum_{\kappa =0}^{\infty }a_{\kappa }\;D_{z,\delta }^{q}\;\left\{z^{\kappa }\right\}.$$

*Therefore, by Lemma 5.1, the desired outcome is acquired.*

**Theorem 22.** *Let* l−1<R(q)<l *,* l∈N *, and let* Q(z) *be an analytic function with its expansion stated by* $Q\left(z\right)=\sum_{\kappa =0}^{\infty }a_{\kappa }\;z^{\kappa }$ *in* |z|<r (r>0) *, then*

(59) $$\;D_{z,\delta }^{q}\left\{Q\left(z\right)\right\}=\sum_{\kappa =0}^{\infty }a_{\kappa }\;D_{z,\delta }^{q}\;\left\{z^{\kappa }\right\}\;=z^{-q}\sum_{\kappa =0}^{\infty }a_{\kappa }\;\frac{\Gamma \left(\kappa +1\right)\;}{\Gamma \left(\kappa -q+1\right)\;}\;\frac{B_{\delta ,\varrho ,\eta }^{\tau ,\mu }\left(\kappa +1,\;l-q\right)}{B\left(\kappa +1,\;l-q\right)}z^{\kappa }\;.\;$$

*Proof. Using (54) to the function* Q(z) *, it leads*

(60) $$D_{z,\delta }^{q}\left\{Q\left(z\right)\right\}=D_{z,\delta }^{q}\left\{\sum_{\kappa =1}^{\infty }a_{\kappa }\;z^{\kappa }\right\}=\frac{1}{\Gamma \left(l-q\right)}\int_{0}^{z}\sum_{\kappa =0}^{\infty }a_{\kappa }\;{℘}^{\kappa }{\left(z-℘\right)}^{l-q-1}\;\psi_{\varrho ,\eta }^{\tau ,\mu }\left(\frac{-\delta z^{2}}{℘\left(z-℘\right)}\right)d℘$$

*Since the series is uniformly convergent in* |z|<r *and the integral is convergent, the order of summation and integration can be interchangeable. Thus,* Eq. (60) *reduces to*

$$D_{z,\delta }^{q}\left\{Q\left(z\right)\right\}=\sum_{\kappa =0}^{\infty }a_{\kappa }\left\{\frac{1}{\Gamma \left(l-q\right)}\int_{0}^{z}{℘}^{\kappa }\;{\left(z-℘\right)}^{l-q-1}\;\psi_{\varrho ,\eta }^{\tau ,\mu }\left(\frac{-\delta z^{2}}{℘\left(z-℘\right)}\right)\right\}=\sum_{\kappa =0}^{\infty }a_{\kappa }\;D_{z,\delta }^{q}\;\left\{z^{\kappa }\right\}.$$

*Therefore, using Lemma 5.2 attains the desired outcome.*

*The following outcome is realized from Lemma 5.1 and Theorems 5.1.*

**Theorem 23.** *Let* R(q)<0 *,* 0<R(ℷ) *, and let* Q(z) *be an analytic function with its expansion stated by* $Q\left(z\right)=\sum_{\kappa =0}^{\infty }a_{\kappa }\;z^{\kappa }$ *in* |z|<r (r>0) *, then*

(61) $$D_{z,\delta }^{q}\left\{z^{ℷ-1}\;Q\left(z\right)\right\}=\sum_{\kappa =0}^{\infty }a_{\kappa }\;D_{z,\delta }^{q}\;\left\{z^{ℷ+\kappa -1}\right\}\;=\frac{\;z^{ℷ-q-1}}{\Gamma \left(-q\right)}\sum_{\kappa =0}^{\infty }a_{\kappa }\;B_{\delta ,\varrho ,\eta }^{\tau ,\mu }\left(ℷ+\kappa ,\;-q\right)\;z^{\kappa }\;.$$

*The following outcome is realized from Lemma 2 and Theorems 22.*

**Theorem 24.** *Let* l−1<R(ℷ−q)<l<R(ℷ) *, where* l∈N *, and let* Q(z) *be an analytic function with its expansion stated by* $Q\left(z\right)=\sum_{\kappa =0}^{\infty }a_{\kappa }\;z^{\kappa }$ *in* |z|<r (r>0) *, then*

(62) $$\begin{array}{cc} D_{z,\delta }^{q}\left\{z^{ℷ-1}\;Q\left(z\right)\right\} & =\sum_{\kappa =0}^{\infty }a_{\kappa }\;D_{z,\delta }^{q}\;\left\{z^{\kappa +ℷ-1}\right\}\\ & =z^{ℷ-q-1}\sum_{\kappa =0}^{\infty }a_{\kappa }\;\frac{\;\Gamma \left(ℷ+\kappa \right)\;B_{\delta ,\varrho ,\eta }^{\tau ,\mu }\left(ℷ+\kappa ,\;l-q\right)}{\Gamma \left(ℷ-q+\kappa \right)\;B\left(ℷ+\kappa ,\;l-q\right)}z^{\kappa }. \end{array}$$

*Moreover,*

(63) $$\;D_{z,\delta }^{q}\left\{z^{ℷ-1}\;Q\left(z\right)\right\}=\frac{\Gamma \left(ℷ\right)z^{ℷ-q-1}}{\Gamma \left(ℷ-q\right)}\sum_{\kappa =0}^{\infty }a_{\kappa }\frac{{\left(ℷ\right)}_{\kappa }\;B_{\delta ,\varrho ,\eta }^{\tau ,\mu }\left(ℷ+\kappa ,\;l-q\right)\;}{{\left(ℷ-q\right)}_{\kappa }\;B\left(ℷ+\kappa ,\;l-q\right)}\;z^{\kappa }\;.$$

**Theorem 25.** *For* J∈C *,* R(q)<0 *,* 0<R(ℷ) *and* |z|<1 *, then*

(64) $$D_{z,\delta }^{ℷ-q}\left\{z^{ℷ-1}\;{\left(1-z\right)}^{-J}\right\}=\frac{\Gamma \left(ℷ\right)\;z^{q-1}}{\Gamma \left(q\right)}\;F_{\delta ,\varrho ,\eta }^{\tau ,\mu }\left(J,\;ℷ;q;z\right)\;.\;$$

*Proof. In view of (53), it follows that*

(65) $$D_{z,\delta }^{ℷ-q}\left\{z^{ℷ-1}\;{\left(1-z\right)}^{-J}\right\}=\frac{1}{\Gamma \left(q-ℷ\right)}\int_{0}^{z}{℘}^{ℷ-1}\;{\left(1-℘\right)}^{-J}{\left(z-℘\right)}^{q-ℷ-1}\;\psi_{\varrho ,\eta }^{\tau ,\mu }\left(\frac{-\delta z^{2}}{℘\left(z-℘\right)}\right)d℘.$$

*Setting* ℘=zν *in (65) and by (40), it yields*

$$D_{z,\delta }^{ℷ-q}\left\{z^{ℷ-1}\;{\left(1-z\right)}^{-J}\right\}=\frac{z^{q-1}}{\Gamma \left(q-ℷ\right)}\int_{0}^{1}\nu^{ℷ-1}\;{\left(1-\nu \right)}^{q-ℷ-1}\;{\left(1-z\nu \right)}^{-J}\;\psi_{\varrho ,\eta }^{\tau ,\mu }\left(\frac{-\delta }{\nu \left(1-\nu \right)}\right)d\nu .$$

$$=\frac{B\left(ℷ,q-ℷ\right)\;z^{q-1}}{\Gamma \left(q-ℷ\right)}\;F_{\delta ,\varrho ,\eta }^{\tau ,\mu }\left(J,\;ℷ;q;z\right)=\frac{\Gamma \left(ℷ\right)\;z^{q-1}}{\Gamma \left(q\right)}\;F_{\delta ,\varrho ,\eta }^{\tau ,\mu }\left(J,\;ℷ;q;z\right).$$

**Theorem 26.** *For* J∈C *,* l−1<R(ℷ−q)<l<R(ℷ) *, where* l∈N *, and* |z|<1 *, then*

(66) $$D_{z,\delta }^{ℷ-q}\left\{z^{ℷ-1}\;{\left(1-z\right)}^{-J}\right\}=\frac{\Gamma \left(ℷ\right)}{\Gamma \left(q\right)}z^{q-1}\;F_{\delta ,\varrho ,\eta }^{\tau ,\mu }\left(J,\;ℷ;l+q;z\right)$$

*Proof. From (54), it follows that*

(67) $$D_{z,\delta }^{ℷ-q}\left\{z^{ℷ-1}\;{\left(1-z\right)}^{-J}\right\}=\frac{d^{l}}{dz^{l}}D_{z,\delta }^{ℷ-q-l}\left\{z^{ℷ-1}\;{\left(1-z\right)}^{-J}\right\}=\frac{d^{l}}{dz^{l}}\left\{\frac{1}{\Gamma \left(l+q-ℷ\right)}\int_{0}^{z}{℘}^{ℷ-1}{\left(1-℘\right)}^{-J}{\left(z-℘\right)}^{l+q-ℷ-1}\psi_{\varrho ,\eta }^{\tau ,\mu }\left(\frac{-\delta z^{2}}{℘\left(z-℘\right)}\right)d℘\right\}.$$

*Setting* ℘=zν *in (67) and by (40), it yields*

$$D_{z,\delta }^{ℷ-q}\left\{z^{ℷ-1}\;{\left(1-z\right)}^{-J}\right\}=$$

$$\frac{d^{l}}{dz^{l}}\left\{\frac{1}{\Gamma \left(l+q-ℷ\right)}\int_{0}^{1}\nu^{ℷ-1}{\left(1-z\nu \right)}^{-J}z^{l+q-ℷ+ℷ-1}{\left(1-\nu \right)}^{l+q-ℷ-1}\psi_{\varrho ,\eta }^{\tau ,\mu }\left(\frac{-\delta }{\nu \left(1-\nu \right)}\right)d\nu \right\}$$

$$=\left(\frac{d^{l}}{dz^{l}}z^{l+q-1}\right)\left\{\frac{1}{\Gamma \left(l+q-ℷ\right)}\int_{0}^{1}\nu^{ℷ-1}{\left(1-z\nu \right)}^{-J}\;{\left(1-\nu \right)}^{l+q-ℷ-1}\psi_{\varrho ,\eta }^{\tau ,\mu }\left(\frac{-\delta }{\nu \left(1-\nu \right)}\right)d\nu \right\}$$

$$=\frac{\Gamma \left(l+q\right)}{\Gamma \left(q\right)}z^{q-1}\frac{B\left(ℷ,l+q-ℷ\right)\;}{\Gamma \left(l+q-ℷ\right)}\;F_{\delta ,\varrho ,\eta }^{\tau ,\mu }\left(J,\;ℷ;l+q;z\right)=\frac{\Gamma \left(ℷ\right)}{\Gamma \left(q\right)}z^{q-1}\;F_{\delta ,\varrho ,\eta }^{\tau ,\mu }\left(J,\;ℷ;l+q;z\right)$$

*Consequently, Theorems 25 and 26 yield the following outcomes.*

**Theorem 27.** *For* J∈C *,* R(q)<0 *,* 0<R(ℷ) *and* |z|<min{1,|1−ω|} *, then*

(68) $$\sum_{\kappa =0}^{\infty }\frac{\;{\left(ℷ\right)}_{\kappa }}{\kappa !}\omega^{\kappa }\;F_{\delta ,\varrho ,\eta }^{\tau ,\mu }\left(J+\kappa ,\;ℷ;q;z\right)={\left(1-z\right)}^{-J}\;F_{\delta ,\varrho ,\eta }^{\tau ,\mu }\left(J,\;ℷ;q;\frac{z}{1-\omega }\right).$$

*Proof. Considering the following elementary identity*

$${\left[\left(1-z\right)-\omega \right]}^{-J}={\left(1-\omega \right)}^{-J}{\left(1-\frac{z}{1-\omega }\right)}^{-J},$$

*and expand* ${\left[(1-z)-\omega \right]}^{-J}$ *to gain*

(69) $${\left(1-z\right)}^{-J}\sum_{\kappa =0}^{\infty }\frac{\;{\left(ℷ\right)}_{\kappa }}{\kappa !}{\left(\frac{\omega }{1-z}\right)}^{\kappa }\;={\left(1-\omega \right)}^{-J}{\left(1-\frac{z}{1-\omega }\right)}^{-J}\;.$$

*Multiplying* Eq. (69) *by* $z^{ℷ-1}$ *and employing the new RL-fractional derivative operator* $D_{z,\delta }^{ℷ-q}$ *on* Eq. (69) *, we yield*

$$D_{z,\delta }^{ℷ-q}\left\{\sum_{\kappa =0}^{\infty }\frac{\;{\left(ℷ\right)}_{\kappa }}{\kappa !}\;z^{ℷ-1}{\left(1-z\right)}^{-J-\kappa }\;\right\}=D_{z,\delta }^{ℷ-q}\left\{{\left(1-\omega \right)}^{-J}\;z^{ℷ-1}{\left(1-\frac{z}{1-\omega }\right)}^{-J}\right\}.$$

*Due to the uniform convergence of the implicated series, the order can be interchanged, and using Theorem 25 leads to*

$$\sum_{\kappa =0}^{\infty }\frac{\;{\left(ℷ\right)}_{\kappa }}{\kappa !}\omega^{\kappa }D_{z,\delta }^{ℷ-q}\left\{z^{ℷ-1}\;{\left(1-z\right)}^{-J-\kappa }\right\}={\left(1-\omega \right)}^{-J}\;D_{z,\delta }^{ℷ-q}\left\{\;z^{ℷ-1}{\left(1-\frac{z}{1-\omega }\right)}^{-J}\right\}=\;{\left(1-\omega \right)}^{-J}\;F_{\delta ,\varrho ,\eta }^{\tau ,\mu }\left(J,\;ℷ;q;\frac{z}{1-\omega }\right)\;.$$

**Theorem 28.** *For* J∈C *,* l−1<R(ℷ−q)<l<R(ℷ) *, where* l∈N *, and* |z|<min{1,|1−ω|} *, then*

(70) $$\sum_{\kappa =0}^{\infty }\frac{\;{\left(ℷ\right)}_{\kappa }}{\kappa !}\omega^{\kappa }\;F_{\delta ,\varrho ,\eta }^{\tau ,\mu }\left(J+\kappa ,ℷ;l+q;z\right)={\left(1-z\right)}^{-J}\;F_{\delta ,\varrho ,\eta }^{\tau ,\mu }\left(J,\;ℷ;l+q;\frac{z}{1-\omega }\right).$$

*Proof. Analogous to the preceding Theorem 5.7 and by using Theorem 5.6, we derive the desired outcome.*

## Conclusion

This paper presents a sophisticated extension of Beta functions using Mittag-Leffler and Hurwitz-Lerch Zeta functions as kernels. The study uses convolution to come up with complete analytical formulations that include Mellin transforms, integral representations, functional and summation relations, and derivative formulae. There is a lot of mathematical and statistical value in the suggested extended Beta function and its uses, especially when it comes to special functions and fractional calculus. As this approach improves the comprehension and use of Beta distributions in academic and practical contexts, potential future applications of these functions are in statistical physics, fractional calculus, and advanced probability modelling. Also, they could expand on current functions for wider uses and create new mathematical tools.

## Limitations

Not applicable.

## Ethics statements

The platforms’ data redistribution policies were complied with.

## Funding statement

This research received no external funding.

## CRediT authorship contribution statement

**Faten F. Abdulnabi:** Writing – original draft. **Hiba F. Al-Janaby:** Writing – review & editing. **Firas Ghanim:** Writing – review & editing.

## Declaration of competing interest

The authors declare that they have no known competing financial interests or personal relationships that could have appeared to influence the work reported in this paper.

## Data Availability

No data was used for the research described in the article.
